# Carbon-Based MOF Derivatives: Emerging Efficient Electromagnetic Wave Absorption Agents

**DOI:** 10.1007/s40820-021-00658-8

**Published:** 2021-06-06

**Authors:** Xue Zhang, Jing Qiao, Yanyan Jiang, Fenglong Wang, Xuelei Tian, Zhou Wang, Lili Wu, Wei Liu, Jiurong Liu

**Affiliations:** 1 School of Materials Science and Engineering, Shandong University, Jinan, 250061 People’s Republic of China; 2 State Key Laboratory of Crystal Materials, Shandong University, Jinan, 250100 People’s Republic of China

**Keywords:** Carbon-based MOF derivatives, Special structures, Multiple attenuation mechanisms, Impedance matching, EMW absorption

## Abstract

To tackle the aggravating electromagnetic wave (EMW) pollution issues, high-efficiency EMW absorption materials are urgently explored. Metal–organic framework (MOF) derivatives have been intensively investigated for EMW absorption due to the distinctive components and structures, which is expected to satisfy diverse application requirements. The extensive developments on MOF derivatives demonstrate its significantly important role in this research area. Particularly, MOF derivatives deliver huge performance superiorities in light weight, broad bandwidth, and robust loss capacity, which are attributed to the outstanding impedance matching, multiple attenuation mechanisms, and destructive interference effect. Herein, we summarized the relevant theories and evaluation methods, and categorized the state-of-the-art research progresses on MOF derivatives in EMW absorption field. In spite of lots of challenges to face, MOF derivatives have illuminated infinite potentials for further development as EMW absorption materials.

## Highlights

- In terms of components and structures, this review summarizes progresses and highlights strategies of MOF derivatives for efficient electromagnetic wave absorption.
- We also systematically delineate relevant theories and points out the prospects and current challenges.

## Graphical abstract

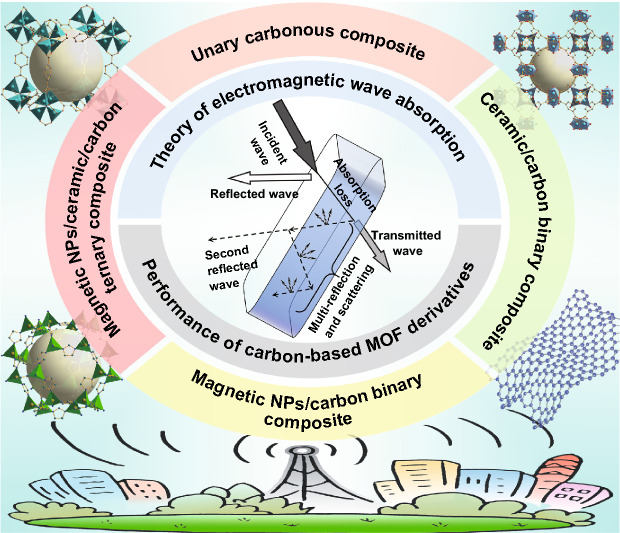

## Introduction

Owing to the extensive utilization of the communication devices working in GHz, the electromagnetic environment has been deteriorating. The redundant electromagnetic waves emitted from various electronic facilities can seriously threaten the physical health and disturb normal equipment operation [1, 2]. To solve these problems, electromagnetic wave (EMW) absorption materials are highly desired [3, 4]. Ideal EMW absorption materials should possess the characteristics of light weight, strong absorption, broad bandwidth, and thin matching thickness to meet diverse application requirements [5, 6]. Thus, various EMW absorption materials have been widely explored currently. Nevertheless, some challenges still exist, such as the insufficient effective absorption abilities in S band and the deficient research about K band. More significantly, the thickness of absorbers is relatively thick and the effective absorption bandwidth (EAB) is still narrow on the whole view, which is not conductive enough to the practical application.

In terms of EMW attenuation mechanisms, EMW absorption materials can be generally divided into conductive materials, dielectric materials, and magnetic materials [7–9]. Typical conductive absorption materials are mainly carbonous, such as graphene, carbon nanotubes (CNTs), and carbon nanofibers [10–13]. And they exhibit obvious advantages in low density and high stability [14, 15]. Nevertheless, the impedance mismatching impedes their further application [16]. Inorganic ceramics and semiconductors are the representatives of dielectric materials, possessing a remarkable superiority in thermal stability [17–19]. However, low attenuation capacity restricts the enhancement of absorption intensity. Magnetic metal and ferrites as typical magnetic absorption substances are frequently used to reduce the matching thickness [20–22]. However, the high density and inherent impedance mismatching as their inevitable shortage limits their further applications. Therefore, to overcome the weakness of single-component EMW absorption materials, the design and configuration of multi-component have been becoming the inevitable choice.

**Fig. 1:**
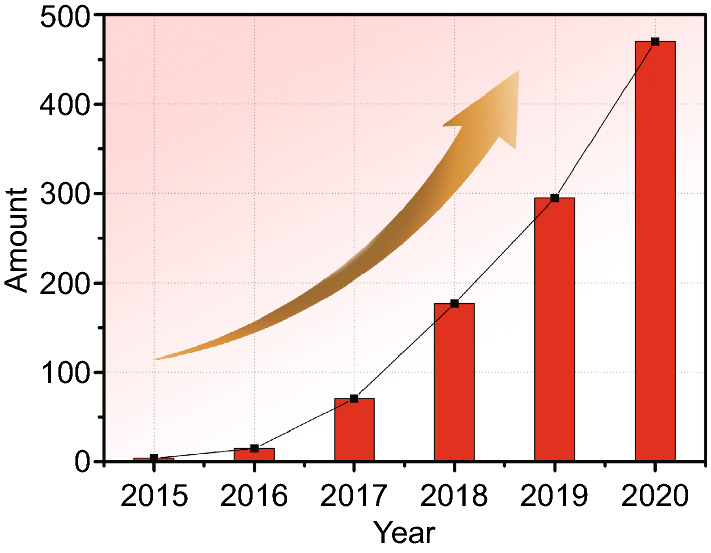
Number of published items on carbon-based MOF derivatives as EMW absorption materials from 2015 to 2020

Metal–organic frameworks (MOFs), as a kind of novel multi-functional materials, were constructed by metallic ion and organic ligand bonded though coordination [23–25]. The common MOFs contain Isoreticular Metal–Organic Framework (IRMOF), zeolitic imidazolate framework (ZIF), Coordination Pillared-Layer (CPL), Materials of Institute Lavoisier (MIL), Porous Coordination Network (PCN), University of Oslo (UiO), and so on [26–33]. Due to their big specific surface area, high porosity, and special periodic structure, in recent years, MOFs have jumped into the limelight and been widely applied in gas separation, medicines, catalysis, and so on [34–38]. Besides, taking MOFs as building blocks to develop porous carbon-based MOF derivatives with diversiform features such as controllable defects, adjustable structures, and alterable compositions has also drawn attention from researchers who devoted to EMW absorption [39, 40]. As shown in Fig. 1, since the first reported in 2015, MOF derivatives for EMW absorption have a rapid development in the following years. The nature of carbon-based MOF derivatives can be elaborately designed by modulating the cabonization condition and collocating the metallic ion and organic ligand in precursors [41]. Simultaneously, combining MOF derivatives with other functional materials offers more opportunities [42].

Compared with other absorbers, on the one hand, carbon-based MOF derivatives possess high conductivity, facilitating strong conductive loss. With carbonization at different temperature, the degree of graphitization can be flexibly adjusted, which would further regulate the conductive loss. And the terminal groups of carbon matrix can induce the dipole polarization. On the other hand, carbon-based MOF derivatives possess a prominent characteristic of high dispersion, by which the metal oxide, metal nanoparticles, or other components can be highly dispersed into carbon matrix to create large heterogeneous phase interfaces to significantly enhance the interfacial polarization loss and optimize the impedance matching conditions. Moreover, carbonous components are endowed with low density, high mechanical strength, and high stability, which is beneficial to decrease the density of nanocomposite and improve environmental adaptability. All these advantages of carbon-based MOF derivatives are extremely attractive for the exploration of ideal EMW absorption materials. And so many relevant reports have demonstrated that they can be deservedly regarded as the most promising choice.

**Fig. 2:**
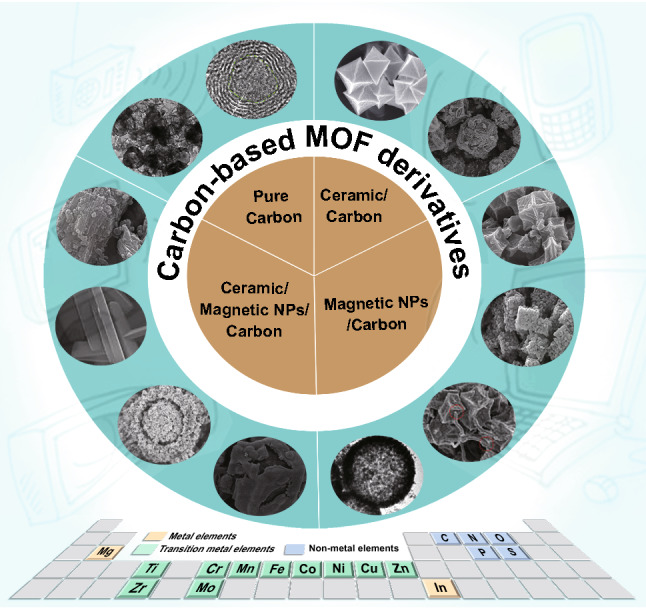
Carbon-based MOF derivatives for EMW absorption applications. Reproduced with permission from Refs. [43–53]. And the corresponding elements in the reported carbon-based MOF derivatives for EMW absorption

In this review, we comprehensively summarized the theory of EMW absorption mechanism and the recent researches about carbon-based MOF derivatives as EMW absorption materials. As shown in Fig. 2, in terms of the composition variations, carbon-based MOF derivatives used for EMW absorption can be classified into four categories: unary carbonous materials, ceramic/carbon binary composites, magnetic NPs/carbon binary composites, and magnetic NPs/ceramic/carbon ternary composites. And the bottom of the picture is the corresponding constituent elements of the aforementioned nanocomposites. The specific performances, internal mechanisms and application scenarios were thoroughly illustrated by typical examples. Finally, the current challenges for carbon-based MOF-derived EMW absorption materials are pointed out, and the perspectives on future development directions are expected.

## Theories of Electromagnetic Wave Absorption

### Macroscopical Description

**Fig. 3:**
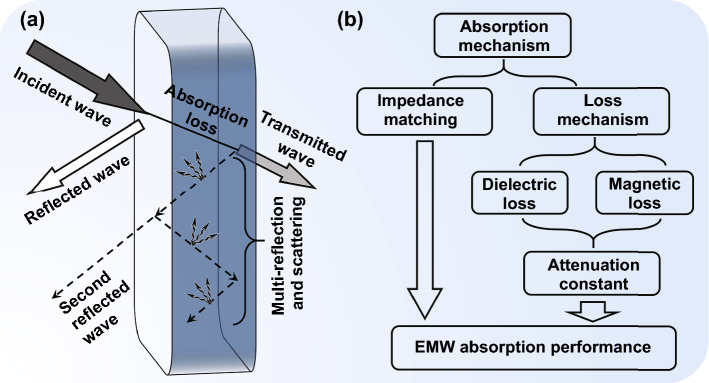
**a** Propagation of EMWs in absorption materials. **b** Schematic diagram of EMW absorption mechanisms

As shown in Fig. 3a, the incident EMWs radiated onto the material surface can be divided into three parts: the reflected, the dissipated, and the transmitted [54, 55]. On the basis of the plane wave model, all transmitted waves will be reflected on the absorber–metal surface [56]. In this condition, EMW absorption materials require the reflected waves are reduced and the dissipated energy is enhanced as much as possible. The most essential two parameters to determine the EMW absorption performance are the impedance matching condition and attenuation capacity [57]. Impedance matching condition adjudicates whether the incident waves can enter into the absorber. An ideal impedance matching condition means the incident waves can totally enter into the absorber and no waves will be reflected on the air-absorber surface. The attenuation capacity decides whether the EMWs inside the absorber can be dissipated. Excellent attenuation capacity requires a strong energy conversion and dissipation ability, which means the EMW energy can be transformed into thermal energy in various methods. Only if both the two preconditions are achieved, a good EMW absorption performance can be obtained.

### Performance Evaluation

All of the absorption performances are finally determined by the electromagnetic parameters: complex permittivity (*ε*_r_ = *ε′ − jε″*) and complex permeability (*μ*_r_ = *μ′ − jμ″*), whose real part stands for energy storage and imaginary part represents energy dissipation [58]. Usually, the EMW absorption performances are calculated on the basis of transmission line theory [59]. And the reflection loss (*RL*) was applied to evaluate the EMW absorption performances. A smaller *RL* value means lesser reflected waves and stronger absorption intensity. And the *RL* value of − 10 dB refers to 90% of incident EMWs can be converted into thermal energy. Correspondingly, the frequency range in which *RL* value is smaller than − 10 dB stands for the effective absorption bandwidth [60]. On the basis of transmission line theory, the specific calculation process from electromagnetic parameters to RL is expressed as the followed formulas [61, 62]:

1 $$Z_{{{\text{in}}}} = Z_{0} \sqrt {\frac{{\mu_{{\text{r}}} }}{{\varepsilon_{{\text{r}}} }}} \tanh \left[ {\frac{2\pi jfd}{{\text{c}}}\sqrt {\mu_{{\text{r}}} \varepsilon_{{\text{r}}} } } \right]$$

2 $${\text{RL = 20log}}\left| {\frac{{(Z_{{{\text{in}}}} - Z_{0} )}}{{(Z_{{{\text{in}}}} + Z_{0} )}}} \right|$$

where *f* is the frequency, *d* is the matching thickness, *c* is the velocity of light in vacuum, and *Z*_0_ and *Z*_in_ are the free space impedance and the input impedance of absorbers, respectively.

In some cases, the *RL* is replaced by the reflection coefficient (*R*) to directly evaluate the EMW absorption capacities [63]. Similarly, a smaller *R* value stands for stronger absorption ability, and the *R* value of the EMW black body is zero.

3 $$R = \left| {\frac{{Z_{{{\text{in}}}} - Z_{0} }}{{Z_{{{\text{in}}}} + Z_{0} }}} \right|$$

### Impedance Matching

To achieve a strong absorption intensity (a minimum of *RL* or *R* value), according to the equations mentioned above, the value of (*Z*_in_ − *Z*_0_) should be equal to 0, namely *Z*_in_/*Z*_0_ = 1 [64]. This result tells us that an excellent impedance matching condition requires the input impedance of absorbers (*Z*_in_) is as close to free space impedance (*Z*_0_) as possible. Considering that *Z*_in_ and *Z*_0_ cannot maintain consistent in both real and imaginary part, in some calculation, the criterion of |*Z*_in_/*Z*_0_|= 1 is employed to displace *Z*_in_/*Z*_0_ = 1. Thus, when the value of |*Z*_in_/*Z*_0_| is equal to 1, the absorber can obtain a perfect impedance matching condition to make sure the incident EMWs can enter into the absorber as much as possible.

4 $$\left| {Z_{{{\text{in}}}} {/}Z_{0} } \right| = \left| {\sqrt {\frac{{\mu_{{\text{r}}} }}{{\varepsilon_{{\text{r}}} }}} \tanh \left[ {\frac{2\pi jfd}{{\text{c}}}\sqrt {\mu_{{\text{r}}} \varepsilon_{{\text{r}}} } } \right]} \right|$$

In some reports, the delta function can be also applied to access the impedance matching degree, which can be calculated by the followed equation [65, 66]:

5 $$\left| \Delta \right|{ = }\left| {\sinh^{2} \left( {Kfd} \right) - M} \right|$$

where *K* and *M* could be determined by the relative complex permittivity and permeability:

6 $$K = \frac{{4\pi \sqrt {\mu^{\prime}\varepsilon^{\prime}} \sin \frac{{\delta_{e} + \delta_{m} }}{2}}}{{c\cos \delta_{e} \cos \delta_{m} }}$$

7 $$M = \frac{{4\mu^{\prime}\cos \delta_{e} \varepsilon^{\prime}\cos \delta_{m} }}{{\left( {\mu^{\prime}\cos \delta_{e} - \varepsilon^{\prime}\cos \delta_{m} } \right)^{2} + \left[ {\tan \left( {\frac{{\delta_{m} }}{2} - \frac{{\delta_{e} }}{2}} \right)} \right]^{2} \left( {\mu^{\prime}\cos \delta_{e} { + }\varepsilon^{\prime}\cos \delta_{m} } \right)^{2} }}$$

where *δ*_e_ and *δ*_m_ refer to the electromagnetic parameters *μ*_r_/*ε*_r_ and the matching thickness *d*/λ, respectively. The value of delta equaled to 0 indicates a perfect impedance matching. And the incident waves will not be reflected on the surface of absorber.

The criterion of |*Z*_in_/*Z*_0_| was widely applied by virtue of its convenience and universality. But in some cases, even if the value of |*Z*_in_/*Z*_0_| is equal to 1, the values of *Z*_in_ and *Z*_0_ are far apart, which would result in the criterion ineffective. |Δ| is a relatively complex criterion of impedance matching. Nevertheless, during the derivation of the |Δ|, the values of *Z*_in_ and *Z*_0_ were considered approximate, which would avoid the criteria losing efficacy.

### Interference Cancellation Principle

To some extent, the concepts of interference cancellation principle and impedance matching overlap and complement with each other. The impedance matching condition is based on equivalent circuit theories while the interference cancellation principle focuses on the specific and microcosmic behaviors of EMWs. Both the two concepts delineate whether or not the electromagnetic wave can enter the absorber and no reflected waves. If the EMWs reflected from air–absorber interface are out of phase by 180° to those reflected from absorber–backboard interface, the destructive interference leads to the disappearance of reflected waves, which seems like all EMWs are trapped inside the absorber to form standing waves [67, 68]. This concept is also called quarter-wavelength model, by which we can calculate the theoretical optimum thickness for a given material at any frequency [69, 70]:

8 $$d_{{\text{m}}} = \frac{nc}{{4f_{{\text{m}}} \sqrt {\left| {\varepsilon_{{\text{r}}} \mu_{{\text{r}}} } \right|} }},(n = 1,3,5 \cdots )$$

where *d*_m_ stands for the matching thickness; *f*_m_ is the matching frequency. On many reports, this equation is used to compare whether experiment matching thickness is consistent with the theoretical matching thickness, further to expound whether the performance results accord with the interference cancellation principle.

### Attenuation Capacity

The attenuation mechanisms of EMW absorption materials are divided into two aspects: dielectric loss and magnetic loss (Fig. 3b). And the dielectric loss mainly includes the conductive loss and polarization–relaxation loss. Generally, the dielectric loss tangent (tan*δ*_E_ = *ε*″/*ε*′) and magnetic loss tangent (tan*δ*_M_ = *μ*″/*μ*′) are used to assess the capacity of dielectric loss and magnetic loss [71, 72].

Conductive loss refers to that the alternating electric field induces microcurrent to convert electric field energy into heat. Specifically, for highly conductive materials, when EMWs enter into the interior of the absorber, the electrons and holes will move under the excitation of electric field force to form the electric currents. Due to the existence of resistance, the currents will generate heat to consume electrical energy, by which finally the attenuation of EMW is realized. According to the free electron theory, for highly conductive materials the conductivity (*σ*) can directly affect the imaginary part of permittivity (*ε*″) [73]:

9 $$\varepsilon^{\prime\prime} \approx \sigma /2\pi \varepsilon_{0} f$$

where *ε*_0_ is the permittivity of vacuum. Thus, a larger conductivity will result in a higher *ε*″ value, which can enhance the conductive loss.

Polarization–relaxation loss is originated from the lossy polarization–relaxation process. Specifically, if the polarization–relaxation process can keep up with the changes of low-frequency alternating electric field, this process is lossless. In high frequency range, if the polarization–relaxation process cannot catch up with the changes of alternating electric field, this process will be lossy. Typical, polarization–relaxation processes include the interfacial polarization–relaxation, dipole polarization–relaxation, ionic polarization–relaxation, and electronic polarization–relaxation [74–76]. The interfacial polarization–relaxation is attributed to the heterogeneous charge distribution in defects, heterojunctions, and phase interfaces [77]. The dipole polarization–relaxation is mainly ascribed to the deflection or displacement of dipoles such as polar molecules and functional groups under the induction of alternating [78, 79]. However, ionic and electronic polarization–relaxation usually take place at much higher frequency (10^3^–10^6^ GHz), so that they are always excluded [80, 81]. According to the Debye theory, if the conductivity is negligible, for a polarization–relaxation process, the complex permittivity will satisfy the following equations [82–84]:

10 $$\varepsilon_{{\text{r}}}^{\prime } = \varepsilon_{\infty } + \frac{{\varepsilon_{{\text{s}}} - \varepsilon_{\infty } }}{{1 + \left( {2\pi f} \right)^{2} \tau^{2} }}$$

11 $$\varepsilon_{{\text{r}}}^{\prime \prime } = \frac{{2\pi f\tau \left( {\varepsilon_{{\text{s}}} - \varepsilon_{\infty } } \right)}}{{1 + \left( {2\pi f} \right)^{2} \tau^{2} }}$$

where *ε*_∞_ represents the permittivity at high frequency limit; *ε*_s_ refers to the static permittivity; and *τ* is the polarization–relaxation time.

And subsequently the followed equation can be deduced by combing the above two equations:

12 $$\left( {\varepsilon_{{\text{r}}}^{\prime } - \frac{{\varepsilon_{{\text{s}}} + \varepsilon_{\infty } }}{2}} \right)^{2} + \left( {\varepsilon_{{\text{r}}}^{\prime \prime } } \right)^{2} = \left( {\frac{{\varepsilon_{{\text{s}}} - \varepsilon_{\infty } }}{2}} \right)^{2}$$

In mathematics, there is a semicircle whose radius is $\frac{{\varepsilon_{{\text{s}}} - \varepsilon_{\infty } }}{2}$ and center is ($\frac{{\varepsilon_{{\text{s}}} + \varepsilon_{\infty } }}{2}$, 0) on the *ε*′–*ε*″ curve, which is usually called Cole–Cole plot [85]. And each semicircle represents one polarization–relaxation process.

However, in the actual analysis, the Cole–Cole plot can hardly delineate a standard semicircle due to the conductivity. Thus, Eq. (11) should be modified to Eq. (13) if the conductivity is considered [86].

13 $$\varepsilon_{{\text{r}}}^{\prime \prime } = \frac{{2\pi f\tau \left( {\varepsilon_{{\text{s}}} - \varepsilon_{\infty } } \right)}}{{1 + \left( {2\pi f} \right)^{2} \tau^{2} }} + \frac{\sigma }{{\varepsilon_{0} \omega }}$$

Thus, Eq. 12 could be evolved into Eq. 14 as followed:

14 $$\left( {\varepsilon_{{\text{r}}}^{\prime } - \frac{{\varepsilon_{{\text{s}}} + \varepsilon_{\infty } }}{2}} \right)^{2} + \left( {\varepsilon_{{\text{r}}}^{\prime \prime } - \frac{\sigma }{{\varepsilon_{0} \omega }}} \right)^{2} = \left( {\frac{{\varepsilon_{{\text{s}}} - \varepsilon_{\infty } }}{2}} \right)^{2}$$

The center coordinate was changed into ($\frac{{\varepsilon_{{\text{s}}} + \varepsilon_{\infty } }}{2}$,$\frac{\sigma }{{\varepsilon_{0} \omega }}$). And this is a moving point, which results in the right end of semicircles turning to the upper right like a “tail” [52]. Thus, on Cole–Cole plots, the slope of the tail can be used to estimate the effect of conductive loss.

Magnetic loss is mainly originated from natural resonance, exchange resonance, eddy current loss, magnetic hysteresis, and domain wall resonance [87, 88]. The magnetic hysteresis is negligible in EMW absorption analysis because the weak field is not strong enough to induce irreversible magnetization. The domain wall resonance usually occurs at much lower frequency rather than the discussed GHz frequency range [89]. To evaluate the contribution of eddy current, the criterion of *C*_0_ is usually utilized.

15 $$C_{0} = \mu^{\prime\prime}\left( {\mu^{\prime}} \right)^{ - 2} f^{ - 1} = 2\pi \sigma \mu_{0} d^{2}$$

where *μ*_0_ is the permeability of the vacuum and d is the thickness of absorber. The value of *C*_0_ will express as a constant, if the magnetic loss is mainly originated from eddy current loss [90, 91].

The natural resonances result from the ferromagnet maximally absorb the energy of alternating magnetic fields, which are usually regarded as the most important magnetic attenuation mechanism in high frequency. The essence of natural resonances is the damping motion of the magnetic moment around the magnetic anisotropy field. The natural resonances effect can be described as the following equations [92–94]:

16 $$2\pi f_{{\text{r}}} = \gamma H_{\alpha }$$

17 $$H_{\alpha } = \frac{{4\left| {K_{1} } \right|}}{{3\mu_{0} M_{{\text{s}}} }}$$

where *f*_r_ is the natural resonance frequency; *γ* is the gyromagnetic ratio; *H*_α_ is the anisotropy energy; *K*_1_ is the anisotropy coefficient; *μ*_0_ is the permeability of free space; and *M*_s_ is the saturation magnetization. Thus, the natural resonance frequency is significantly decided by the anisotropy energy, and further affected by shape anisotropy, magneto-crystalline anisotropy, coercivity, particle size, and so on.

In the meantime, Snoek’s limit restricts the application of the magnetic materials in higher frequency range. The Snoek’s limit can be described as followed equation for isotropic ferromagnetic materials [95–97]:

18 $$(\mu_{i} - 1)f_{r} = \frac{1}{3\pi }\gamma M_{s}$$

where *γM*_s_ represents the Snoek constant; and *γ* and *M*_s_ represent gyromagnetic ratio and saturation magnetization, respectively; *μ*_i_ is the initial permeability; and *f*_r_ is the natural resonance frequency. For a given ferromagnetic material, the *γ* is a constant and *M*_s_ has a ceiling. Thus, if certain methods are adopted to make *f*_r_ shift to higher frequency range, the *μ*_i_ will be inevitably decreased to result in the reduction of magnetic loss. Hence, the Snoek’s limit restricts the application of ferromagnetic materials in high frequency. The considerable methods to break the Snoek’s limit are choosing soft magnetic metals with higher *M*_s_ values such as Fe, Co, Ni, and relevant alloys or regulating the anisotropic equivalent field such as flak particle design.

In the summary, for a EMW absorption material, all above-mentioned loss capacities can be synthetically evaluated by attenuation constant (*α*) [98, 99].

19 $$\alpha { = }\frac{\sqrt 2 \pi f}{c}\sqrt {(\mu^{\prime\prime}\varepsilon^{\prime\prime} - \mu^{\prime}\varepsilon^{\prime}) + \sqrt {(\mu^{\prime\prime}\varepsilon^{\prime\prime} - \mu^{\prime}\varepsilon^{\prime})^{2} + (\mu^{\prime}\varepsilon^{\prime\prime} + \mu^{\prime\prime}\varepsilon^{\prime})^{2} } }$$

A larger attenuation constant reflected a stronger attenuation capacity. Thus, only in the view of attenuation capacity, according to the equation, *ε*″ and *μ*″ values should be as large as possible to obtain a maximized attenuation constant. However, if the impedance matching condition is also considered, the *ε*″ and *μ*″ values should be controlled in an appropriate range.

## Carbon-Based MOF Derivatives for EMW Absorption Applications

In the whole view, carbon-based MOF derivatives can provide a flexible method to combine dielectric and magnetic components, and the features of big surface areas, tunable compositions, and high porosity make them highly desired as more efficient EMW absorption applications. Benefiting from the frame structures of MOFs, during the pyrolysis process of carbon-based MOF derivatives, the metallic ions can directly be transformed into metallic oxide or metals to combine with carbon matrix, which avoids extra complex reduction process. The oxide ceramics can promote the resistance to corrosion and the magnetic metals can provide magnetic loss capacity. Thus, the collocation with dielectric or magnetic components can effectively supplement the performance shortages of carbon materials. On the other side, the low-density carbonous component not only possesses adequate conductivity to enhance the conductive loss, but also can effectively reduce the quality of absorption materials. The abundant surface terminations such as –OH and C = O in the carbon substrate can significantly strengthen the dipole polarization [100]. Besides, on the one hand, the high surface and pore volume features of carbon provide more active sites for multiple scattering dissipations. On the other hand, according to Maxwell–Wagner theory [101]:

20 $$\varepsilon_{eff}^{MG} = \varepsilon_{1} \frac{{(\varepsilon_{2} + 2\varepsilon_{1} ) + 2p(\varepsilon_{2} - \varepsilon_{1} )}}{{(\varepsilon_{2} + 2\varepsilon_{1} ) - p(\varepsilon_{2} - \varepsilon_{1} )}}$$

where *ε*_1_ and *ε*_2_ represent permittivity in the solid and gas state, respectively; *p* is the volume fraction of gas state in the porous materials, and the porosity can significantly regulate the permittivity to optimize the impedance matching condition.

Therefore, owing to the remarkable chemical and physical properties mentioned above, the carbon-based MOF derivatives epitomize a lighting promising application for high-efficient EMW absorption materials to deal with the electromagnetic environment deterioration. In recent five years, studies using carbon-based MOF derivatives as EMW absorption materials have witnessed rapid development as shown in Table 1. And excluding the under-represented unary carbonous MOF derivatives, the average filling ratios, average matching thickness, average matching frequency (*f*_m_), and average bandwidth value for ceramic/carbon, magnetic NPs/carbon, and magnetic NPs/ceramic/carbon MOF derivatives were calculated, and the results are shown in Table 2. According to the comparison, we could find the magnetic NPs/carbon binary MOF derivatives possessed a much lower average filling ratio and a slightly thinner average matching thickness, indicating their advantages in density decrease of absorbers. The magnetic NPs/ceramic/carbon ternary MOF derivatives were equipped with a relatively lower matching frequency, implying they may get an upper hand in low-frequency absorption. Besides, the ternary MOF derivatives obtained a significant enhancement in absorption bandwidth performance. However, we could also notice that a comprehensive performance advantage is hard to achieve, and much exploration of high-performance EMW absorption materials remains to be done.

**Table 1:** EMW absorption performances of various carbon-based MOF derivatives

Type	Composite (precursor)	Filling ratio (wt%)	Minimum RL value (dB)	*f*_m_ (GHz)	Matching thickness (mm)	Bandwidth			Refs
						thickness (mm)	Value (GHz)	Range (GHz)	
Unary carbonous MOF derivatives	S-GA (Fe_2_Ni MIL-88 nanorods and HCl acid corrosion)	10	− 46.2	10.44	2.65	2.0	5.2	12.8–18.0	[52]
	HGS@PAC (CoZn-MOF and HF acid corrosion)	10	− 32.43	9.19	3.7	3.7	4.2	8.2–12.4	[53]
	hollow carbon microcubes (ZIF-67@SiO_2_ and etching)	30	− 60.7	6.9	3.2	1.0 − 5.0	14.4	3.6–18.0	[102]
Ceramic/carbon binary MOF derivatives	TiO_2_/C (MIL-125)	40	− 49.6	15.5	1.6	1.6	4.6	13.4–18.0	[103]
	ZnO/N-doped C (ZIF-8)	50	− 39.7	8.5	4.0	4.0	4.3	7.9–12.2	[104]
	ZnO/Ni_3_ZnC_0.7_/5%-CNT (Ni-Zn-MOF/CNT)	10	− 33.2	5.9	4.9	4.9	2.1	4.0–7.1	[105]
	ZnO/C/RGO (ZIF-8/RGO)	40	− 50.5	14.0	2.4	7.4	2.6	9.6–17.0	[106]
	CuO/C (Cu^2+^/ZIF-67)	50	− 57.5	14.9	1.55	1.55	4.7	13.0–17.7	[44]
	ZrO_2_/C (UIO-66)	50	− 58.7	16.8	1.5	1.7	5.5	11.5–17.0	[43]
	MoC/C (Cu/Mo-MOF)	20	− 49.19	9.04	2.6	2.6	3.20	7.8–11.0	[107]
	wheat-like MgO/C (CPO-27-Mg)	30	− 14.93	12.88	2.0	2.0	4.9	9.0–13.9	[108]
	CoS_2_/N-CNTs (ZIF-67/ sulfuration)	50	− 65.0	16.4	1.6	1.6	4.2	13.8–18.0	[109]
	carbon cloths@CoS_2_/C (carbon cloths@ZIF-67)	30	− 59.6	9.1	2.8	2.5	9.2	8.8–18.0	[110]
Magnetic NPs/carbon binary MOF derivatives	Co/C (MOF-74)	30	− 62.1	11.85	2.4	2.4	4.6	10.1–14.7	[111]
	Co/C (ZnCo-MOF)	20	− 51.6	17.1	1.6	1.6	3.5	14.5–18.0	[112]
	Co/C (CPT-1-Co)	30	− 15.7	15.1	1.7	1.7	5.4	12.3–17.7	[113]
	Co/C (Co(INA)_2_)	33	− 47.6	14.5	2.0	2.0	5.1	12.7–17.2	[114]
	Co/C (ZIF-67)	40	− 35.3	5.8	4.0	2.5	5.8	8.4–14.2	[45]
	Co/CNTs (ZIF-67)	30	− 49.16	14.3	2.5	2.5	4.2	12.4–16.6	[115]
	hollow Co/C (ZIF-67/cetyltrimethylammonium bromide)	30	− 66.5	17.6	1.53	1.0–5.0	14.3	3.7–18.0	[116]
	Co–C/CNTs (ZIF-67/CNTs)	15	− 48.9	9.0	2.99	2.99	3.4	7.7–11.1	[46]
	Co/C (Co NPs/ZIF-67)	25	− 30.31	11.03	3.0	3.0	4.93	8.31–13.24	[117]
	Co/C (Co_3_[HCOO]_6_·DMF/ glucose)	50	− 19.86	5.0	1.6	3.8	3.84	4.2–8.04	[118]
	Co/C (melamine foam@ZIF-67)	20	− 59.82	12.90	2.3	2.1	5.64	12.36–18.0	[119]
	Co/N-CNTs/C (melamine–formaldehyde sponge@ZIF-67)	10	− 51.2	12.0	2.2	2.2	4.1	10.3–14.4	[120]
	CNT/Co/C (ZIF-67/cotton)	10	− 53.5	7.8	2.9	2.0	8.02	9.98–18.0	[121]
	Co/C (GO/PVP/ZIF-67)	10	− 50.7	9.6	2.9	2.9	4.6	8.6–13.2	[122]
	Co/N-decorated C/CNTs (CoZn-ZIF-L)	20	− 15.37	15.49	1.5	1.5	4.5	13.5–18.0	[123]
	N-doped Co/C/CNTs (CoZn-ZIF/CNTs)	25	− 50.0	10.5	2.4	2.5	3.6	8.2–11.8	[124]
	CoZn/N-doped C (CoZn-ZIF)	30	− 53.8	17.6	1.5	2.0	5.3	10.0–15.3	[125]
	Fe/C (Prussian blue)	40	− 22.6	15.0	2.0	2.0	5.3	12.7–18.0	[40]
	Fe/C (Fe-MIL-88A)	40	− 52.9	11.68	3.07	3.07	4.64	9.44–14.08	[126]
	Fe/C (MIL-101-Fe)	5	− 59.2	5.4	4.32	1.8	5.0	13.0–18.0	[127]
	Fe/C (Fe^2+^/ZIF-8)	15	− 29.5	17.2	2.5	3.0	4.3	13.7–18.0	[128]
	Ni wrapped C (Ni(bdc)(ted)_0.5_)	40	− 51.8	10.44	2.6	1.9	4.68	12.82–17.5	[129]
	Ni/C (Ni-ZIF)	40	− 86.8	13.2	2.7	1.5 − 4.0	7.4	4.0–11.4	[130]
	hollow Ni/C (hollow Ni-MOF)	30	− 57.25	16.1	1.8	1.8	5.1	12.9–18.0	[47]
	waxberry-like Ni/C (Ni-btc)	50	− 73.2	12.3	2.2	1.8	4.8	13.2–18.0	[131]
	CoFe@C (CoFe-MOF-74)	10	− 61.8	12.7	2.8	2.8	9.2	8.8–18.0	[132]
	FeCo/C (Fe_3_O_4_ modified ZIF-67)	50	− 21.7	15.2	1.2	1.2	5.8	12.2–18.0	[133]
	FeCo/C/GO (Fe-doped ZIF-67/rGO)	25	− 43.26	11.28	2.5	2.5	9.12	8.88–18.0	[134]
	FeCo/C (Fe_3_O_4_/ZIF-67@wood carbon)	15	− 47.6	15.7	1.5	1.96	8.9	9.1–18.0	[135]
	CoFe@C/rGO (Fe^3+^/ZnCo-MOF/rGO)	10	− 36.08	13.01	3.0	3.5	5.17	8.72–13.89	[136]
	CoFe@C/CNTs ((Fe^3+^/ZnCo-MOF/CNTs))	10	− 40.00	9.86	3.0	2.0	5.62	12.38–18.0	[136]
	CoNi/C (ZIF-67@NiCo-LDH)	10	− 61.02	13.68	2	2	5.2	/	[137]
	CoNi@NC/rGO (CoNi-BTC/rGO)	25	− 68.0	10.9	3.1	2.5	6.7	11.3–18.0	[138]
	Ni_1−x_Co_x_@Carbon (NiCo-MOF)	25	− 59.5	6.0	4.5	2.5	4.7	9.9–14.6	[139]
	NiCo/C (NiCo-MOFs)	30	− 51	17.9	1.5	1.5	4.5	13.5–18.0	[140]
	FeNi@CNT/CNRs (melamine/FeNi-MIL-88B)	25	− 47.0	10.0	2.3	1.6	4.5	13.5–18.0	[141]
	NiFe@C/GO (NiFe PB/GO)	30	− 51.0	7.7	2.8	2.2	3.97	8.3–12.27	[42]
	hollow FeCoNi@C (hollow FeCoNi-MOF-74)	38	− 64.75	15.44	2.1	2.47	8.08	9.92–18.8	[142]
	Fe_3_O_4_@C (Fe-MOF)	40	− 65.5	9.8	3.0	3.0	4.5	7.9–12.4	[143]
Magnetic NPs/ceramic/carbon ternary MOF derivatives	Co/ZnO/C (CoZn-MOF)	30	− 52.6	12.1	3.0	3.0	4.9	10.1–15.0	[50]
	ZnO/Fe/Fe_3_C/C (Fe^III^-MOF-5)	60	− 50.5	7.44	2.6	1.5	4.6	12.5–17.1	[144]
	Co/TiO_2_-C (Mxene/ZIF-67)	45	− 41.1	9.0	3.0	3.0	3.04	7.24–10.28	[51]
	Fe&TiO_2_@C (MXene/Fe-MOF)	40	− 51.8	6.6	3	1.6	6.5	11.5–18.0	[145]
	Co@NPC@TiO_2_ (ZIF-67@TiO_2_)	50	− 51.7	13.8	1.65	1.2–5.0	14.7	3.3–18.0	[48]
	Co/C@V_2_O_3_ (ZIF-67@VO_2_)	50	− 40.1	15.2	1.5	1.5	4.64	13.26–17.9	[146]
	ZnO/NPC@Co/NPC (ZIF-8@ZIF-67)	50	− 45.0	12.5	2.2	1.9	4.2	13.8–18.0	[147]
	ZnO@NPC/Co_3_ZnC (ZnO@ZIF8@ZIF67)	40	− 62.9	14.0	2.2	2.2	5.5	11.1–16.6	[148]
	Ni@C@ZnO (Ni-Zn-MOF)	25	− 55.8	10.0	2.5	2.5	4.1	8.0–12.1	[49]
	Co/CuO/C (Co^2+^/ Cu_3_(btc)_2_)	40	− 25.0	13.72	1.95	1.85	5.28	12.3–18.0	[149]
	Ni/NiO/Cu@C (Ni^2+^/ Cu_3_(btc)_2_)	10	− 38.1	14.8	3.2	3.2	1.5	14.0–15.5	[150]
	Co/ZrO_2_/C (Co^2+^/UIO-66)	50	− 57.2	15.8	3.3	4.6	11.9	6.1–18.0	[151]
	Co/N/C@MnO_2_ (ZIF-67@ polydopamine@MnO_2_)	15	− 58.9	11.1	3.7	3.7	5.6	8.8–14.4	[152]
	CoFe@C@MnO_2_ (CoFe-Prussian blue and MnO_2_ coating)	50	− 64.0	15.6	1.3	1.6	9.2	8.8–18	[153]
	Prussian blue@MoS_2_	40	− 42.83	16.46	2.1	2.5	7.44	9.82–17.26	[154]
	Co–C@Co_9_S_8_ (ZIF-67 and sulfuration)	30	− 54.02	3.04	4.89	2.2	8.2	9.8–18.0	[155]
	SiC@ZIF-67	10	− 40.0	5.0	5.0	2.0	4.84	13.16–18.0	[156]

**Table 2:** EMW absorption performance comparison of different types of carbon-based MOF derivatives

Type	Average filling ratio (wt%)	Average *f*_m_ (GHz)	Average matching thickness (mm)	Average bandwidth value (GHz)
Ceramic/carbon binary MOF derivatives	37.0	12.3	2.5	4.5
Magnetic NPs/carbon binary MOF derivatives	26.4	12.2	2.4	5.6
Magnetic NPs/ceramic/carbon ternary MOF derivatives	37.4	11.5	2.8	6.2

### Unary Carbonous MOF Derivatives

For carbon-based MOF derivatives, the content and conductivity of carbon matrix play the particularly important roles in EMW absorption [157]. The introduction of other functional components would increase the density of the composites, which goes against the original intention of developing lightweight EMW absorption materials. Therefore, some researchers open a new pathway by designing unary carbonous MOF derivatives.

**Fig. 4:**
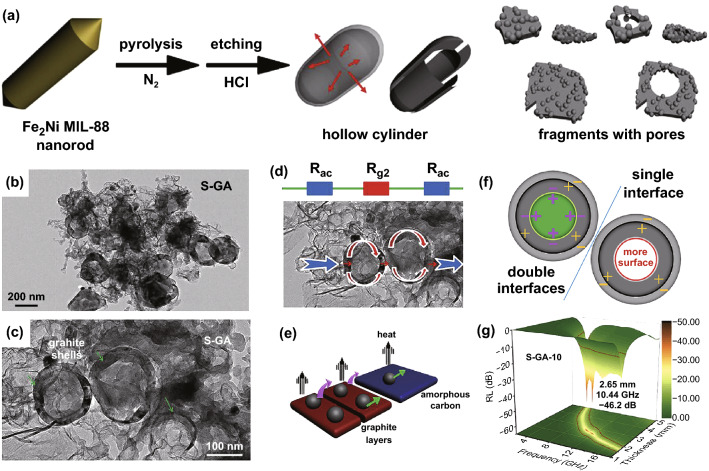
**a** Schematic illustration of fabrication of S-GA. **b, c** TEM image of S-GA. **d–f** Schematic illustration of attenuation of S-GA. **g** 3D RL values of S-GA. Reproduced with permission from Ref. [52]. Copyright © 2018 Elsevier BV

Liu et al. constructed a unique structure, hollow graphite spheres embedded in porous amorphous carbon matrices (S-GA) by etching Fe_3_Ni/C composites derived from Fe_2_Ni MIL-88 nanorods (Fig. 4a–c) [52]. The *RL* value (Fig. 4g) reached − 46.2 dB at 10.44 GHz with a matching thickness of 2.65 mm. And the maximum EAB was achieved in 12.8–18.0 GHz with an ultra-low filling ratio of 10 wt%. The satisfying filling ratio came from the high conductive loss of the graphite carbon (Fig. 4d, e). And the polarization loss (Fig. 4f) between the graphite shells and amorphous carbon matrix also played an auxiliary role in EMW dissipation. Similarly, Xu et al. fabricated a special structure that hollow graphene nano-spheres uniformly confined in porous amorphous carbon particles (HGS@PAC) by HF acid corroding the Co/C composites that pyrolyzed from CoZn-MOFs [53]. The carbon nanocomposite with only 10 wt% loading content exhibited a minimum *RL* value of − 32.43 dB at 9.19 GHz with a thickness of 3.70 mm, corresponding to an EAB of 4.2 GHz. Besides, Zhao et al. prepared hierarchically hollow porous carbon microcubes (HPCMCs) with different structure from ZIF-67@SiO_2_ [102]. The SiO_2_ layer could prevent from shrinkage of MOFs precursors during pyrolysis to obtain various structures carbon materials. After subsequent HF acid etching, the HPCMCs were obtained. At 6.9 GHz, the minimum RL value of HPCMCs could reach − 60.7 dB with the thickness of 3.2 mm. An EAB of 14.4 GHz (3.6–18.0 GHz) was achieved within the thickness rang of 1.0–5.0 mm.

We can conclude that MOF-derived pure carbon with distinctive structures can actually decline the density on the basis of excellent EMW absorption performances. Thus, this kind of MOF derivatives could be an advisable choice for lightweight EMW absorption materials. However, pure carbon with high conductivity could easily result in impedance mismatching. Thus, the introduction of component with low conductivity is essential.

### Ceramic/Carbon Binary MOF Derivatives

Metal oxide, carbide, or sulfide such as TiO_2_, ZnO, CuO, ZrO_2_, MgO, and CoS_2_ possess high chemical and thermal stability, but the low EMW loss capacity impedes its individual application [43, 44, 103, 107, 108, 158]. However, if combined with carbon, these ceramic materials can effectively optimize the impedance mismatching caused by over-high conductivity carbon to achieve favorable absorption performances.

TiO_2_ and ZnO as typical semiconductive transition metal oxides have been widely used as EMW absorption material because of the appropriate band gap, environmental friendliness, and abundance [159]. Nanoporous TiO_2_/carbon nanocomposites fabricated by the direct pyrolysis of MIL-125(Ti) showed a minimum *RL* value of − 49.6 dB at the matching thickness of 1.6 mm, corresponding to a broad EAB of 4.6 GHz [103]. Wu et al. reported ZnO/N-doped porous carbon composites by the thermal treatment of ZIF-8 [104]. The maximum *RL* value can reach − 39.7 dB at the thickness of 4.0 mm with an EAB of 4.3 GHz. Liang et al. prepared ZnO/nanoporous carbon/RGO. In the synthesis process, the ZnO/C was obtained by the pyrolysis of ZIF-8, and through hydrothermal method, the target nanocomposite was gained [106]. The nanocomposite delivered a minimum RL value of − 50.5 dB with the thickness of 2.4 mm. And the EAB of 7.4 GHz was achieved at the thickness of 2.6 mm.

**Fig. 5:**
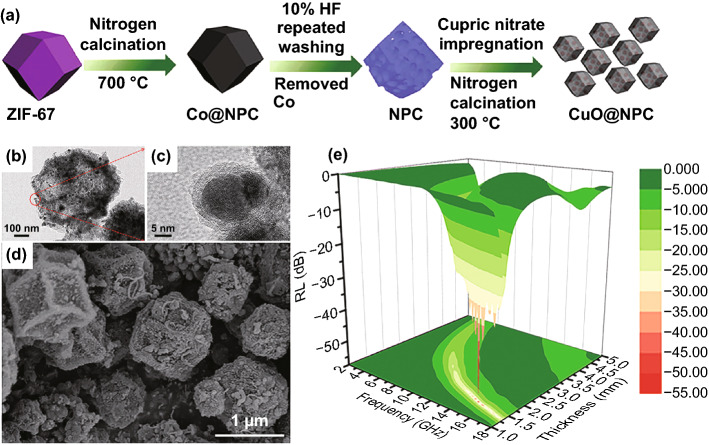
**a** Schematic illustration of the fabrication of the CuO@NPC. **b–d** TEM and SEM images of CuO@NPC. **e** 3D RL map of CuO@NPC. Reproduced with permission from Ref. [44]. Copyright © 2016 Royal Society of Chemistry

Besides, due to its wave-transparent characteristic, CuO is also introduced into EMW absorption materials to optimize the impedance matching. As shown in Fig. 5a–d, Ma et al. prepared ZIF-67 derived nanoporous CuO/carbon composite by etching the metallic Co and soaking the Cu(NO_3_)_2_ into the carbon [44]. The minimum *RL* value of the composite (Fig. 5e) can achieve − 57.5 dB at 14.9 GHz with the thickness of 1.55 mm due to the excellent impedance matching. The EAB (*RL* ≤  − 10 dB) can reach 4.7 GHz. The authors claimed that the valence state of Cu can effectively tune the electromagnetic parameters to affect the absorption properties. For instance, with the introduction of metallic Cu, the value of *ε*″ would be sharply lifted due to the increasing conductivity so that further dissipation could be expected. Besides, the polarization from the heterostructures played an indispensable role in the absorption property enhancement.

In view of the high corrosion resistant of ZrO_2_, MoC, and MgO, Zhang et al. synthesized octahedral ZrO_2_/C nanocomposite by directly thermal treatment of UIO-66 [43]. Benefiting from the intensified polarization loss and improved impedance matching condition, the EMW absorption intensity with a minimum *RL* value of − 58.7 dB achieved. Simultaneously, the EAB could cover 91.3% (3.4–18.0 GHz) of the measured frequency within the thickness of 1.0–5.0 mm. Dai et al. prepared MoC/C nanocomposite by pyrolyzing the Cu/Mo-MOF and etching Cu [107]. The nanocomposite exhibited a minimum *RL* of –49.19 dB at 2.6 mm and an EAB of 4.56 GHz at 1.70 mm. Also, Quan et al. reported the wheat-like MgO/C by directly pyrolyzing CPO-27-Mg [108]. The nanocomposite showed a minimum *RL* of –14.93 dB at the thickness of 2.0 mm, corresponding to an EAB of 4.9 GHz.

Owing to better electrical conductivity, metal sulfides, such as CoS_2_, have jumped into the limelight. Yan et al. prepared hollow CoS_2_/N-doped CNTs (N-CNTs) by pre-carbonization at 350 °C under Ar and H_2_ mixture atmosphere and sulfuration with sulfur powder at 300 °C under N_2_ atmosphere [109]. Benefitting from improved impedance matching and large specific surface area, the minimum *RL* value of CoS_2_/N-CNTs could achieve –65 dB and the EAB could reach 6.2 GHz with the thickness of 1.6 mm. Besides, Liu et al. reported carbon cloths@N-doped carbon/CoS_2_ by sulfuring carbon cloths@ZIF-67 [110]. The nanocomposite delivered a minimum *RL* value of –59.6 dB at 2.8 mm and a EAB of 9.2 GHz with the thickness of 2.5 mm.

All these findings indicated that the synergistic contributions between carbon with high conductivity and uniformly distributed ceramic materials lead to the enhanced EMW absorption performances. However, there are still some issues such as imperfect impedance matching condition. Thus, to further optimize the compositions and constructions of MOF derivatives is still highly desirable to achieve a better EMW absorption performance.

### Magnetic NPs/Carbon Binary MOF Derivatives

The attenuation capacity can be easily regulated by the degree of graphitization of carbon [40], however, to achieve a perfect impedance matching condition, the enhancement of permeability is also indispensable [160]. Thus, for carbon-based composites, the introduction of magnetic components will help to achieve a better EMW absorption performance. Magnetic NPs, as typical magnetic components, have always been widely utilized for EMW absorption [139, 161]. In the carbon-based MOF derivatives, besides the optimized impedance matching condition, magnetic NPs in the porous carbon can also induce the magnetic loss and agitate the interfacial polarization loss. Furthermore, magnetic NPs can be directly reduced by carbon which can avoid additional reduction step. Thus, MOF-derived magnetic NPs/carbon nanocomposites have been widely applied for EMW absorption.

#### Simple Magnetic MOF Derivatives

**Fig. 6:**
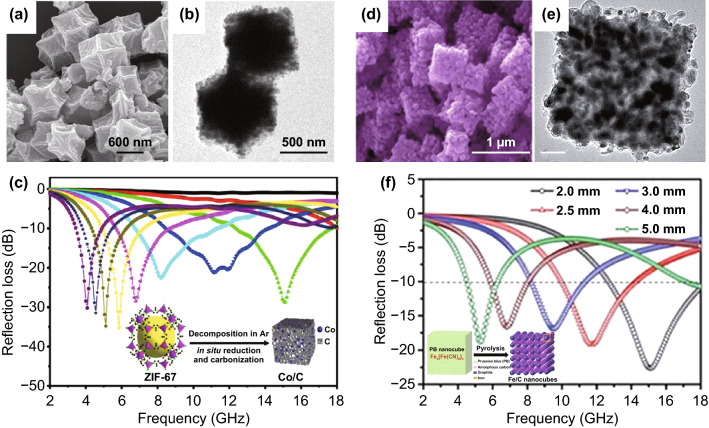
**a, b** SEM and TEM images of Co/C. **c** RL value of Co/C. Reproduced with permission from Ref. [45]. Copyright © 2015 American Chemical Society. **d, e** SEM and TEM images of Fe/C. **f** RL value of Fe/C. Reproduced with permission from Ref. [40]. Copyright © 2015 Royal Society of Chemistry

Some mono-magnetic NPs/carbon nanocomposites with simple shape can be obtained by direct pyrolysis of MOFs. For example, as shown in Fig. 6a, b, Lv et al. fabricated porous Co/C nanocomposites by facile thermal decomposition of ZIF-67 [45]. And the RL value (Fig. 6c) could reach − 35.3 dB with a matching thickness of 4 mm. And an EAB of 5.8 GHz at 2.5 mm could be achieved. The enhanced performance is ascribed to the synergistic effects between the highly porous structure and multiple components. Wang et al. synthesized Co/C nanocomposites by the direct pyrolysis of MOF-74. The minimum *RL* value can reach − 62.12 dB at 11.85 GHz with the thickness of 2.4 mm, corresponding to an EAB of 4.6 GHz [111]. Huang et al. reported Co/C composites by high temperature treatment of ZnCo bimetallic MOF. The minimum *RL* value of the composites achieved − 51.6 dB at a thickness of 1.6 mm [112]. The enhanced absorption performances were mainly attributed to the formation of Co NPs, which greatly improved impedance matching condition. Zhu et al. prepared Co/C composites by directly pyrolyzing a newly-constructed MOF called CPT-1-Co [113]. And the composites carbonized at 700 °C possessed a minimum RL value of − 15.7 dB with an EAB of 5.4 GHz at a ultrathin thickness of 1.7 mm. Li et al. fabricated prismatic Co/C nanocomposite derived from the cubic MOF of [Co(INA)_2_] in which the INA was isonicotinic acid. The nanocomposite pyrolyzed at 650 °C displayed a minimum *RL* value of − 47.6 dB and an EAB of 5.11 GHz at the thickness of 2.0 mm [114]. Ulteriorly, Xiao et al. prepared the Co/CNTs nanohybrid by carbonizing ZIF-67 at the gas flow of C_2_H_2_ and Ar [115]. The *RL* value of nanocomposites could achieve − 49.16 dB at a thickness of 2.5 mm, corresponding to an EAB of 4.2 GHz. Feng et al. synthesized CoZn alloy/N-doped porous carbon nanocomposites derived from CoZn-ZIF [125]. The nanocomposites possessed a minimum *RL* value of − 53.8 dB at 17.6 GHz with a thickness of 1.5 mm. Xu et al. prepared cactus-like hierarchical Co/N-decorated carbon architecture/CNTs nanocomposites by carefully treating CoZn-ZIF-L at 700 °C for 2 h [123]. The products exhibited an optimal absorption performance with a minimum *RL* value of − 44.6 dB at 5.20 GHz, corresponding to a matching thickness of 1.5 mm.

Compared with metallic Co, other magnetic NPs have their unique advantages as well. For example, Fe possesses higher saturation magnetization and magnetic damping capacity [162]. Fe_3_O_4_ holds a better chemical stability [101]. And Ni is superior in reduction condition simplification [163]. Similarly, as shown in Fig. 6d–f, Qiang et al. prepared Fe/C nanocomposites derived from Prussian blue, and Fe/C nanocomposites exhibited an *RL* value of − 22.6 dB at 15.0 GHz at the thickness of 2 mm [40]. Wu et al. also prepared Fe NPs embedded nanoporous carbon by the direct pyrolysis of Fe-MIL-88A [126]. The *RL* value reached − 52.9 dB at 3.07 mm, corresponding to an EAB of 4.64 GHz. Miao et al. reported two isomeric MOFs (MIL-101-Fe with octahedral shape and MIL-88B-Fe with rod shape) derived Fe/C by pyrolysis at Ar atmosphere and studied the effect of morphology on EMW absorption [127]. Attributed to more abundant graphite flakes in MIL-101-Fe-derived Fe/C, the minimum *RL* value could reach − 59.2 dB with a thickness of 4.32 mm, and the EAB could achieve 5 GHz with a thickness of 1.8 mm. Yan et al. also prepared two kind of organic ligands MOF-derived Ni/C, which exhibited a minimum *RL* value of − 86.8 dB at 13.2 GHz with a thickness of 2.7 mm and an EAB of 7.4 GHz with the thickness ranging from 1.5 to 4.0 mm [130]. Xiang et al. prepared porous Fe_3_O_4_@carbon nanocomposites by directly thermal treatment of Fe-MOFs, which exhibited an absorption intensity of − 65.5 dB at 9.8 GHz with a matching thickness of 3.0 mm, corresponding to an EAB of 4.5 GHz [143]. Besides, Liu et al. reported waxberry-like Ni/C microspheres by directly pyrolyzing Ni-btc [131]. The nanocomposite delivered a minimum *RL* value of − 73.2 dB and an EAB of 4.8 GHz at the thickness of 1.8 mm. Liu et al. synthesized porous carbon-wrapped Ni nanocomposites derived from Ni(bdc)(ted)_0.5_. The nanocomposites treated at 500 °C exhibited a minimum *RL* value of − 51.8 dB at a thickness of 2.6 mm, corresponding to an EAB of 3.48 GHz [129]. While the nanocomposites treated at 600 °C possessed a minimum RL value of − 15.0 dB at a thin thickness of 1.8 mm, corresponding to an EAB of 4.72 GHz.

Besides, the construction of hollow structures is also utilized for better EMW absorption performances. The structures are believed to enhance the polarization effect and multi-scattering [164, 165].

**Fig. 7:**
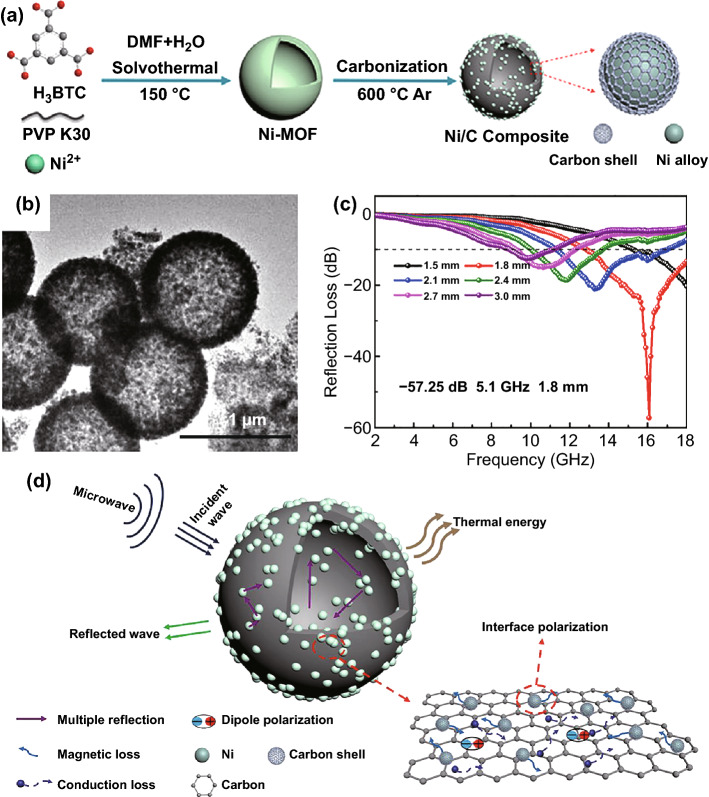
**a, b** Schematic illustration of fabrication and TEM images of Ni/C composites. **c** RL curves and **d** schematic illustration of the possible EMW absorption mechanism of Ni/C composites. Reproduced with permission from Ref. [47]. Copyright © 2020 Elsevier BV

For example, as shown in Fig. 7a, b, Qiu et al. prepared hollow Ni-MOFs precursors by tuning the volume ratio of DMF and H_2_O in solvothermal process, and subsequently, hollow Ni/C microspheres were obtained by pyrolysis [47]. The hollow Ni/C microspheres delivered a maximum *RL* of − 57.25 dB (Fig. 7c) with the matching thickness of only 1.8 mm and corresponding to an EAB of 5.1 GHz. Figure 7d shows that both the hollow structure and the synergistic effect between carbon and nickel nanoparticles contributed to the EMW absorption performance. Moreover, Wang et al. synthesized honeycomb-like Co/C composites pyrolyzed from specially treated ZIF-67 [122]. In the synthesis process, graphene oxide/PVP composites, as the sacrificial template, contributed to transforming ZIF-67 into hierarchically porous structures. The target products obtained a minimum *RL* value of − 50.7 dB and an EAB of 4.6 GHz, while the filler loading is as low as 10 wt%. Furthermore, Li et al. prepared hollow Co/C microspheres derived from ZIF-67 through a one-step template synthesis, in which the cetyltrimethylammonium bromide was used as template [116]. The Co/C nanocomposite delivered a minimum *RL* of − 66.5 dB with the thickness of 1.53 mm at 17.6 GHz. And the EAB could reach 14.3 GHz at the thickness range of 1.0–5.0 mm.

**Fig. 8:**
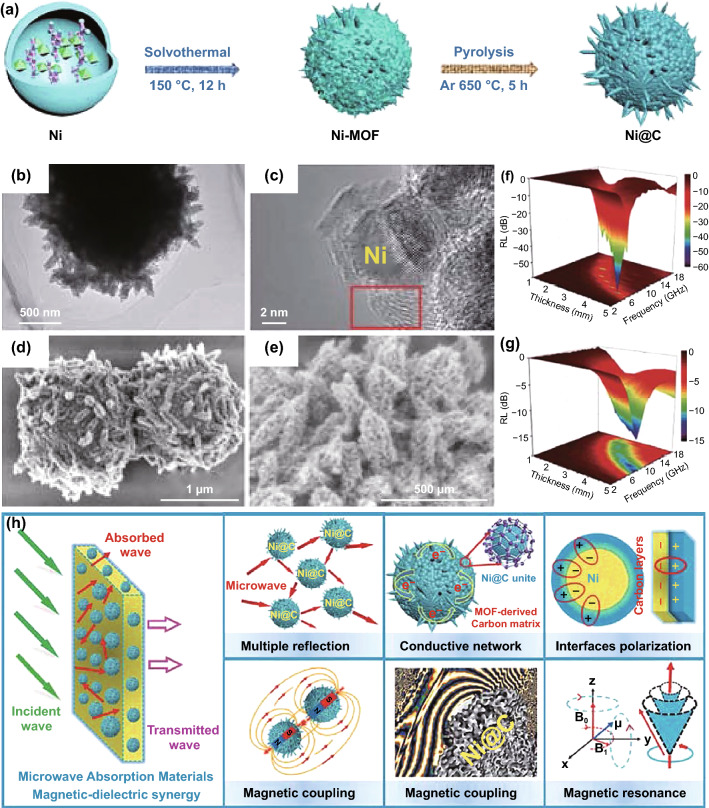
**a** Schematic illustration for the synthetic process of Ni@Carbon microspheres. **b–e** SEM and TEM images of Ni@C composites. **f, g** 3D RL maps of Ni@C composites with different adding mass. **h** Schematic diagram of absorption mechanism. Reproduced with permission from Ref. [139]. Copyright © 2020 Springer

Meanwhile, bi-magnetic NPs/carbon nanocomposites could obtain by the one-step pyrolysis of MOF, which could further enhance the magnetic loss. As shown in Fig. 8, Wang et al. synthesized NiCo-MOF-derived Ni_1−x_Co_x_@Carbon composite and discussed their EMW absorption performances of composites with tunable nano-microstructure [139]. And Ni@C microspheres displayed a minimum *RL* value of − 59.5 dB, corresponding to a EAB of 4.7 GHz. Through the off-axis electron holography, the excellent EMW absorption performance was ascribed to the magnetic-dielectric synergy effect. Xiong et al. reported layered NiCo alloy NPs/nanoporous carbon composite which was obtained by carbonizing NiCo-bimetallic MOFs [140]. The minimum *RL* value of the nanocomposite could achieve − 51 dB at 17.9 GHz, corresponding to an EAB of 4.5 GHz at the thickness of 15 mm. Wang et al. fabricated nest-like CoFe-MOF-derived CoFe@C composite. The minimum *RL* value could achieve − 61.8 dB at 12.7 GHz with the thickness of 2.8 mm, corresponding to an EAB of 9.2 GHz [132]. Moreover, Ouyang et al. constructed trimetallic FeCoNi@C nanocomposite hollow spheres derived from FeCoNi-MOF-74 by adding extra H_2_O in the hydrothermal process [142]. The minimum *RL* value reached − 69.03 dB at 5.52 GHz. And the maximum EAB reached 8.08 GHz (9.92–18 GHz) at the thickness of 2.47 mm.

#### Magnetic MOF Derivatives Combined with Additional Magnetic NPs

The above reports that verified the introduction of magnetic NPs help to optimize impedance matching conditions to obtain a much thinner thickness. However, the simple preparation process, onefold structures, and fixed proportion limit the further application of this kind of MOF derivatives as high-performance EMW absorption materials.

**Fig. 9:**
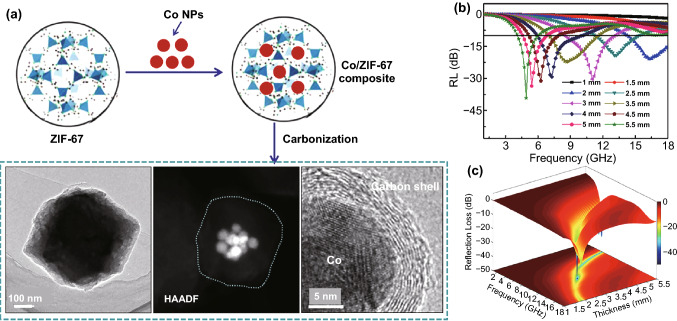
**a** Synthetic route and TEM images of Co NPs/ZIF-67 composites. **b, c** Reflection loss of Co/C-700 with various thicknesses and corresponding 3D RL maps. Reproduced with permission from Ref. [117]. Copyright © 2017 American Chemical Society

Considering that the nature of unchangeable component proportion in certain MOFs may restrain the further enhancement of EMW absorption performances, Wang et al. embedded extra metallic NPs into the MOF derivatives by special treatment [117]. As shown in Fig. 9, the pre-prepared Co NPs were impregnated dispersed into the framework of ZIF-67, and then, the hybrids were pyrolyzed at different temperatures. The obtained Co NPs/porous carbon nanocomposites treated at 700 °C exhibited a minimum *RL* value of − 30.31 dB at 11.03 GHz with a thickness of 3.0 mm. And an EAB of 4.93 GHz can be reached. The nanocomposites treated at 800 °C possessed a minimum *RL* value of − 13.87 dB at 12.9 GHz with a thickness of 2.0 mm, corresponding to an EAB of 3.91 GHz. And Liu et al. reported Fe/C derived from Fe^2+^-encapsulated ZIF-8. The Fe^2+^ ion was first encapsulated in ZIF-8 by grinding, and after carbonization, the Fe/C nanocomposite was obtained [128]. The minimum *RL* value of − 29.5 dB and an EAB of 4.3 GHz were achieved. Zhang et al. prepared FeCo/carbon by thermal decomposition of the additional Fe_3_O_4_ modified ZIF-67 [133]. The minimum RL value of FeCo/C reached − 21.7 at the thickness of 1.2 mm, corresponding to an EAB of 5.8 GHz. These works provided a new idea for designing MOF derivatives with adjustable loading contents. Wang et al. prepared hollow porous CoNi/C composite derived from ZIF-67@NiCo-LDH CSs [137]. The minimum RL value of the nanocomposite could achieve − 61.02 dB at 13.68 GHz, corresponding to an EAB of 5.2 GHz with the thickness of 2 mm.

#### Magnetic MOF Derivatives Combined with Additional Carbon Source

**Fig. 10:**
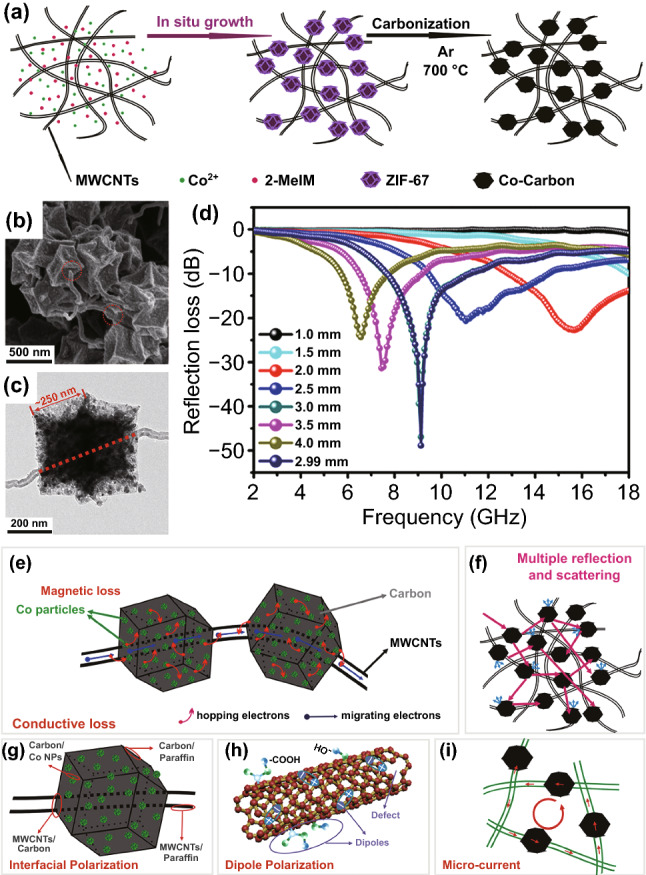
**a** Schematic illustration of the fabrication of Co–C/CNTs composite. **b, c** SEM and TEM images of aligned Co–C/CNTs composite. **d** RL values of Co–C/CNTs composite with different thickness. **e, f** Schematic illustration of EMW absorption mechanism. Reproduced with permission from Ref. [46]. Copyright © 2017 American Chemical Society

CNTs and graphene possess the properties of low density, good chemical stability, and superior electrical conductivity, which can be applied in EMW absorption as the reinforcement elements. Particularly, as shown in Fig. 10, Yin et al. prepared the Co–C/CNTs composite by the carbonization of ZIF-67/CNTs [46]. The conductive loss was significantly improved, which attributed to the combination of CNTs. And combined with other loss mechanisms such as magnetic loss and dielectric loss, the nanocomposite exhibited a minimum *RL* value of − 48.9 dB at the thickness of 2.99 mm with filler loading as low as 15 wt%.

Similarly, Shu et al. synthesized N-doped Co–C/CNTs nanocomposite by carbonizing the CoZn-ZIF/CNTs at 700 °C for 4 h [124]. The minimum *RL* value of the nanocomposite could reach − 50.0 dB at the thickness of 2.4 mm. The EAB could achieve 4.3 GHz at the thickness of 1.8 mm. Moreover, Yang et al. reported MOF-derived porous NiFe@C nanocubes with graphene oxide (GO) combined, which showed a minimum *RL* value of − 51 dB at 7.7 GHz with a thickness of 2.8 mm [42]. The introduction of GO further generated multiple interfaces, which promoted the polarization loss. Wang et al. prepared FeCo/N-doped carbon/rGO by carbonization Fe-doped ZIF-67/rGO. The nanocomposite exhibited a *RL* value of − 43.26 dB with the thickness of 2.5 mm, corresponding to a EAB of 9.12 GHz [134].

**Fig. 11:**
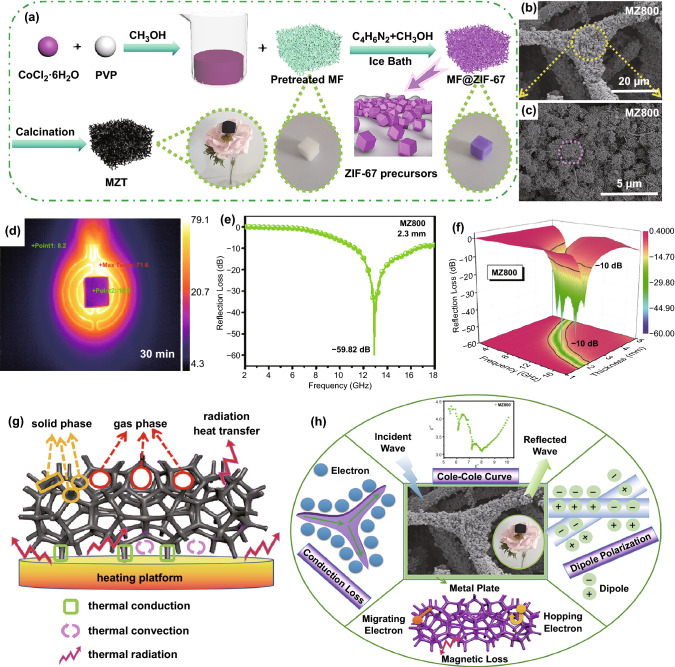
**a** Schematic illustration of the synthesis process of annealed 3D melamine foam@ZIF-67 hybrids (MZT) samples. **b, c** SEM images of MZ800. **d** Thermal infrared images of MZ800 captured at 30 min. **e** Effective bandwidth of MZ800 at 2.3 mm. **f** 3D RL plots of MZ800. **g, h** Schematics of the heat transfer mechanism and EMW absorption mechanisms of the 3D hybrid foam. Reproduced with permission from Ref. [119]. Copyright © 2020 American Chemistry Society

Recently, due to the heat resistance, 3D interconnected network, and high surface area of melamine foam [166], it has been widely applied in EMW absorption. Combined with MOF, the derivatives exhibited outstanding EMW absorption performance. As depicted in Fig. 11, Gu et al. prepared Co/C composites with 3D porous network structure by deriving melamine foam@ZIF-67 [119]. The porosity and conductivity of the products could facilitate the enhancement of dielectric loss. Attributed to the synergistic effect of dielectric loss and magnetic loss, the minimum *RL* value could achieve − 59.82 dB. Moreover, benefited from the enhanced thermal conduction, thermal convection, and thermal radiation, the nanocomposite showed infrared stealth and heat insulation function. Similarly, Yang et al. synthesized Co/N-CNTs/C sponge composite derived from melamine–formaldehyde sponge@ZIF-67 as well. The minimum *RL* value could reach − 51.2 dB and the EAB could up to 4.1 GHz at the thickness of 2.2 mm [120]. Yang et al. reported hierarchical CNT/Co/C fiber derived from ZIF-67/cotton [121]. Due to the hollow fibrous structure and multiple loss mechanism, the nanocomposite exhibited a minimum *RL* value of − 53.5 dB at 7.8 GHz with the thickness of 2.9 mm. And the EAB could reach 8.02 GHz at the thickness of 2 mm. Xiong et al. synthesized FeCo/C nanocages by the pyrolysis of Fe_3_O_4_/ZIF-67@wood carbon [135]. Attributed to the enhanced polarization loss, magnetic coupling loss, and hierarchical conductive network, the nanocomposite was endowed with outstanding EMW absorption performance. The minimum *RL* value could reach − 47.6 dB at 15.7 GHz with the thickness of 1.5 mm, and an EAB of 8.9 GHz achieved at the thickness of 1.96 mm.

Furthermore, Liu et al. reported the sponge-like Co/C nanocomposite, as well as its synthesis procedures, microstructures and absorption performances [118]. By adding extra glucose into Co_3_[HCOO]_6_·DMF precursors, the carbon contents could be noticeably changed. Meanwhile, with more glucose added, in the final products, the microstructure of extra-introduced carbon on sponge-like matrix changed from fragments to vertically aligned nanoflakes and eventually the sponge-like structure transformed into a thick layer with extra fragments. The optimal sponge-like Co/C nanocomposites exhibited a minimum *RL* value of − 19.86 dB at the thickness of 1.6 mm with the EAB of 4.12 GHz. This work provided opportunities for designing MOF derivatives with adjustable carbon matrix contents.

All the above enhanced excellent performances are attributed to the enhanced magnetic loss and the hierarchical hollow designs or other special structure, which can optimize the impedance matching and strengthen the dissipation capacity to improve the final EMW absorption performances. Nonetheless, considering the magnetic NPs are easy to be oxidized or corroded, how to improve the environmental adaptability of relevant composites is still a big challenge.

### Magnetic NPs/Ceramic/Carbon Ternary MOF Derivatives

As aforementioned, carbon, ceramic, and magnetic NPs exhibit their unique advantages in EMW absorption. Thus, hybridization of them to construct magnetic NPs/ceramic/carbon ternary composites may pave the way to further enhance the EMW absorption performance. In this point, how to reasonably collocate compositions and construct microstructures to achieve the synergistic effects is particularly important. Nevertheless, the finite species for EMW absorption confine the research potential in collocating compositions, so that the researchers devoted more to the construction of microstructures.

#### One-step Synthesis of Ternary MOF Derivatives

In Liao’s work, Co/ZnO/C microrods were directly derived from CoZn-MOF by pyrolysis. The minimum *RL* value could be as low as − 52.6 dB at 12.1 GHz with a matching thickness of 3.0 mm and an EAB of 4.9 GHz [50]. The absorption property is mainly originated from the production of abundant loss pathways, including conductive loss, dielectric loss, and magnetic loss. Liu et al. prepared ZnO/Fe/Fe_3_C/carbon (ZFC) composite by the direct pyrolysis of Fe^III^-MOF-5 [144]. The composite treated at 700 °C exhibited an absorption performance with a minimum RL value of − 30.4 dB at a thickness of 1.5 mm, corresponding to an EAB of 4.6 GHz.

#### Two-step Synthesis of Ternary MOF Derivatives

**Fig. 12:**
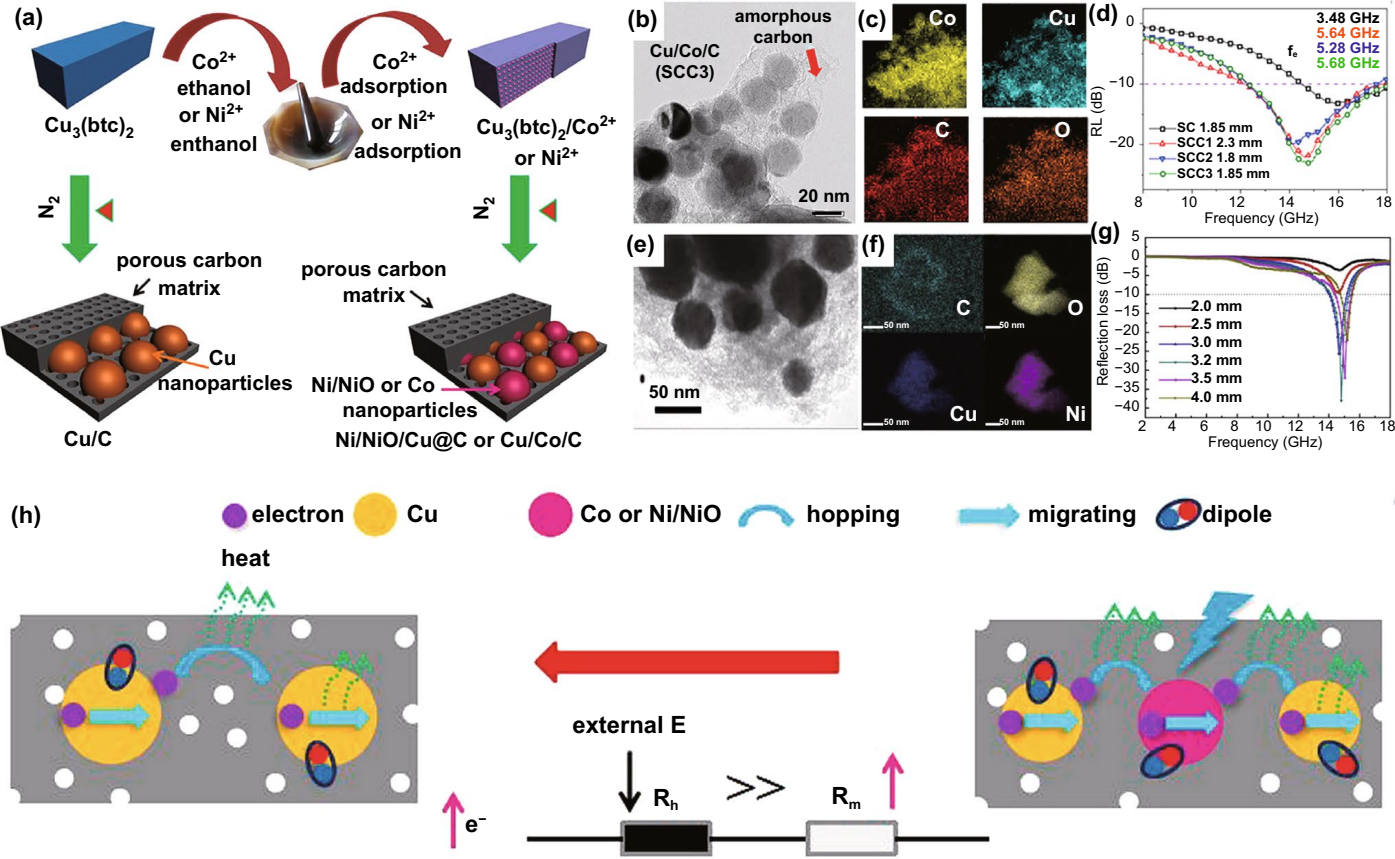
**a** Formation of porous Cu/C, Cu/Co/C and Ni/NiO/Cu@C composite. **b–d** TEM images and elemental mapping and RL values of Cu/Co/C composite. **e–g** TEM images and elemental mapping and RL values of Ni/NiO/Cu@C composite. **h** Possible attenuation mechanisms and equivalent circuit mode of Cu/Co/C and Ni/NiO/Cu@C composite. **a–d, h** Reproduced with permission from Ref. [149]. Copyright © 2018 American Chemistry Society. **e–g** Reproduced with permission from Ref. [150]. Copyright © 2019 Elsevier BV

However, the method of direct treatment for bimetallic MOFs cannot realize the random collocation of components. Thus, extra treating processes such as mixing, dipping, and cladding are widely applied. Liu et al. synthesized the Co/CuO/C nanocomposite [149]. As shown in Fig. 12a, by thoroughly grinding of Cu-MOFs (Cu_3_(btc)_2_) in a handful of Co^2+^ solutions and subsequent pyrolysis, the Co ions were also encapsulated in the carbon matrix. This method was simple and flexible, and made full use of the porous structures to uniformly disperse Co NPs, which further protect Co NPs from agglomeration. In Fig. 12b, c, the elements of Co and Cu were evenly distributed on the carbon matrix. The composite possessed a minimum *RL* value (Fig. 12d) of − 25.0 dB at 13.72 GHz with a thickness of 1.95 mm, corresponding to an EAB of 5.36 GHz. Analogously, Huang et al. prepared Ni/NiO/Cu@C composite using the same method [150]. And the particles were also well dispersed (Fig. 12e, f). The composite exhibited a minimum *RL* value (Fig. 12g) of − 38.1 dB at a thickness of 3.2 mm with only 10 wt% of filler loading. Figure 12h shows the possible absorption mechanism of the above two nanocomposites, and it can be clarified by modified equivalent circuit mode. Zhang et al. prepared Co/ZrO_2_/C nanocomposite derived from Co^2+^-adsorbed NH_2_-UIO-66 [151]. The product exhibited a minimum *RL* value of − 57.2 dB at 15.8 GHz with a matching thickness of 3.3 mm. The maximum EAB could reach 11.9 GHz at the thickness of 4.6 mm. Wang et al. fabricated Ba_0.85_Sm_0.15_Co_2_Fe_16_O_27_ hexaferrite/Co/porous carbon derived from ferrite/ZIF-67 which exhibited a minimum *RL* value of − 31.05 dB at 8.4 GHz and an EAB of 4.8 GHz at the thickness of 1.5 mm [167].

#### Ternary MOF Derivatives with Special Structures

On this basis, some researchers focused on the design of core–shell structures to achieve performance improvements. For example, Zhang et al. designed a novel core–shell Co@carbon@TiO_2_ nanocomposite [48]. The nanocomposite with high TiO_2_ content exhibited a minimum *RL* value of − 51.7 dB at 1.65 mm, and the *RL* value for the nanocomposite with low TiO_2_ content could reach − 31.7 dB at 1.5 mm. The coating thickness TiO_2_ shell affected the impedance matching condition to enhance the EMW absorption performance, and it could protect the Co core from oxidizing as well. Similarly, Zhou et al. prepared hierarchical Co/C@V_2_O_3_ hollow spheres by pyrolyzing V_2_O_3_ coated ZIF-67, which exhibited the EMW performances with a minimum *RL* value of − 40.1 dB at the matching thickness of 1.5 mm, corresponding to an EAB of 4.64 GHz [146]. Liang et al. further developed a core–shell structure of ZnO/nanoporous carbon@Co/nanoporous carbon (ZnO/NPC@Co/NPC) by the pyrolysis of ZIF-8@ZIF-67 precursor [147]. The core–shell nanocomposites exhibited a minimum *RL* value of − 28.8 dB at 16 GHz with a matching thickness of 1.9 mm and an EAB of 4.2 GHz. Feng et al. also designed a ZnO@N-doped porous carbon/Co_3_ZnC core–shell heterostructures derived from ZnO@ZIF-8@ZIF-67 with minimum *RL* value of − 62.9 dB and broad EAB of 5.5 GHz [148].

**Fig. 13:**
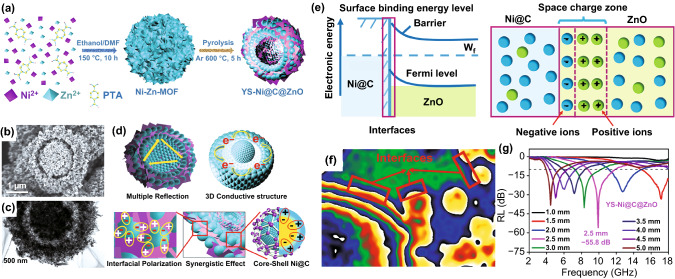
**a** Illustration of the synthetic process of yolk-shell Ni@C@ZnO microspheres. **b, c** SEM and TEM images of Ni@C@ZnO. **d, e** Microwave absorption mechanisms of Ni@C@ZnO. **f, g** Off-axis electron hologram and the RL values of Ni@C@ZnO. Reproduced with permission from Ref. [49]. Copyright © 2020 Elsevier BV

Wang et al. reported the yolk-shell Ni@C@ZnO nanocomposite as shown in Fig. 13a–c [49]. The special core–shell structure accelerated the multiple reflection and enhanced the dipole and interfacial polarization (Fig. 13d, e). And the hologram (Fig. 13f) confirmed the existence of multiple interfaces. As a result, the nanocomposite delivered a minimum *RL* value of − 55.8 dB at 2.5 mm and an EAB bandwidth of 4.1 GHz (Fig. 13g). Wang et al. prepared Co/N/C@MnO_2_ nanocomposite by pyrolysis ZIF-67@polydopamine and coating MnO_2_. The nanocomposite displayed a minimum *RL* value of − 58.9 dB and an EAB of 5.56 GHz at the thickness of 3.7 mm [152]. Similarly, Zhang et al. fabricated CoFe@C@MnO_2_ nanocomposite. Firstly, the CoFe@C was obtained by the carbonization CoFe-Prussian blue. Then, the CoFe@C nanocubes were coated with manganese dioxide by a hydrothermal reaction to obtain the target nanocomposite [153]. CoFe@C@MnO_2_ nanocomposite displayed a minimum *RL* value of − 64 dB at 15.6 GHz with the thickness of 1.3 mm and an EAB of 9.2 GHz at the thickness of 1.6 mm. Furthermore, Zhao et al. reported Prussian blue@MoS_2_ synthesized by solvothermal method [154]. The minimum *RL* value of the nanocomposite could achieve − 42.83 dB with the thickness of 2.1 mm at 16.46 GHz. And the EAB could reach 7.31 GHz at 2.4 mm.

For sulfide or carbide, Liu et al. reported a yolk–shell structured Co–C/Void/Co_9_S_8_ nanocomposite by sulfurating ZIF-67 [155]. The nanocomposite displayed a minimum *RL* value of − 54.02 dB at the loading of 30 wt%, and the EAB could achieve 8.2 GHz with the thickness of 2.2 mm at the loading of 25 wt%. The synergistic effect of abundant heterointerfaces, controlled cavities, and multiple losses facilitated the EMW absorption performance enhancement. Zhang et al. fabricated one-dimensional ZIF-67 loaded SiC through the thermal treatment at 500 °C. The nanocomposite exhibited a minimum *RL* value of − 40 dB with the thickness of 5 mm [156]. And the EAB could reach 4.84 GHz with the thickness of 2.0 mm.

Furthermore, some researchers combined MOF with other current hot materials such as MXenes to get better EMW absorption performances. For example, Liao et al. fabricated Co/TiO_2_-C hybrids which was derived from Ti_3_C_2_T_x_ (MXene)/ZIF-67 [51]. The MXenes possessed 2D multilayered microstructures, which are beneficial for the absorption performances. Thus, the obtained Co/TiO_2_-C hybrids possessed a minimum *RL* value of − 41.1 dB at 9.0 GHz with a thickness of 3.0 mm, corresponding to an EAB of 3.04 GHz.

**Fig. 14:**
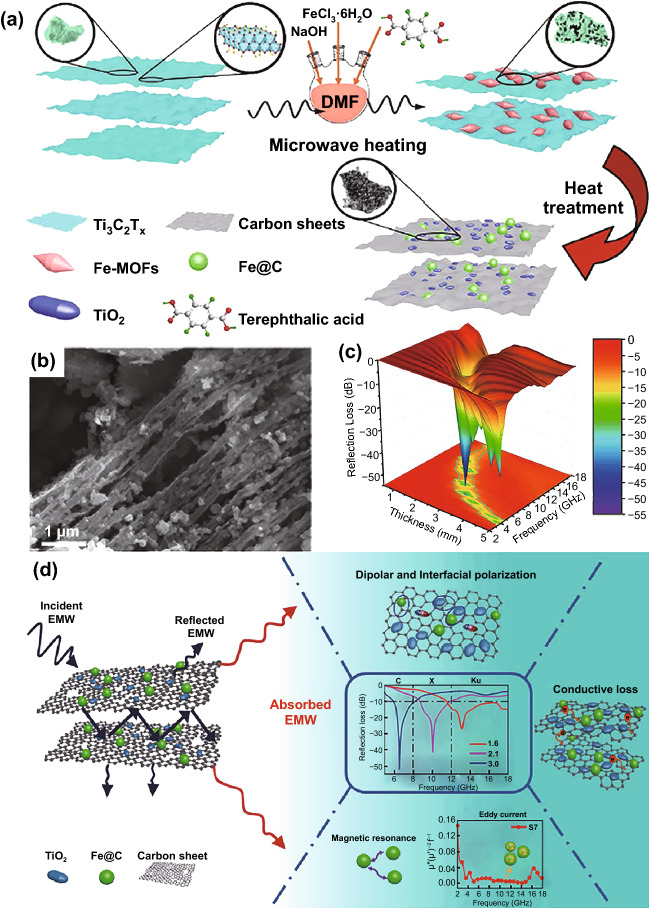
**a** Schematic representation of the facile synthesis route of the Fe&TiO_2_@C. **b, c** SEM image and 3D RL map of Fe&TiO_2_@C. **d** Illustration of EMW absorption mechanisms for Fe&TiO_2_@C. Reproduced with permission from Ref. [145]. Copyright © 2020 Springer

Similarly, Deng et al. prepared sandwich-like Fe&TiO_2_@C nanocomposite by the pyrolysis of MXene/Fe-MOF at H_2_/Ar atmosphere shown in Fig. 14 [145]. The minimum *RL* value could achieve − 51.8 dB at 6.6 GHz at the thickness of 3 mm and the EAB could reach 6.5 GHz at the thickness of 1.6 mm. Particularly, the interfacial polarization between Fe, TiO_2_, and carbon made a great contribution to the EMW absorption performance.

In brief, compared with the binary MOF derivatives, the introduction of both magnetic and ceramic components into carbon can indeed offer more opportunities to control the impedance matching condition, and make full use of various attenuation pathways. Besides, the ternary nanocomposites exhibited a higher tolerance to the harsh environment and a higher degree of controllability. Thus, the strategy of MOF-derived magnetic NPs/ceramic/carbon nanocomposite was expected as a promising pathway for high-efficiency EMW absorption materials. Nevertheless, after summarizing, we found that the EAB of most above-mentioned MOF derivatives is confirmed to 4 GHz. Thus, how to further expand the EAB performance is still a big challenge.

## Conclusions and Prospects

In this review article, we summarized the common theories and the recent progress of carbon-based MOF derivatives in field of EMW absorption. In terms of composition variations, carbon-based MOF derivatives for EMW absorption are classified into four categories: unary carbonous materials, ceramic/carbon binary composites, magnetic NPs/carbon binary composites, and magnetic NPs/ceramic/carbon ternary composites. Abundant reports verified that the basic features of highly porous structures, high dispersion, strong operability, and readily tunable compositions were exploitable for EMW absorption performance promotion. By regulating the pyrolysis conditions, the graphitization degree and porosity of carbon matrix can be adjusted to control the permittivity, as well as the conductive loss. By selecting the categories of constitute units in MOF precursors, the derivatives with expected compositions can be obtained to optimize the electromagnetic parameters. By constructing more elaborate structures or combining with other functional materials, the impedance matching conditions and attenuation capacities can be further improved. All these superiorities make the carbon-based MOF derivatives highly expected to be new-type high-performance EMW absorption materials. Therein, because of the combination of both magnetic loss and dielectric loss, the further optimized impedance matching, as well as a more flexible preparation method, MOF-derived magnetic NPs/carbon binary composites have been studied most extensively. While attributed to a more controllable composition and structure, and a more optimized EMW performance, the magnetic NPs/ceramic/carbon ternary composites are becoming more attractive for researchers.

The carbon-based MOF derivatives bring some new blood into the development of EMW absorption materials. But there are still a lot remaining to be done to meet the continuously developing performance requirement. To achieve a more comprehensive property and further expand the multi-component combination mode, MOF-derived sulfide, phosphide, and nitride composites have been gradually drawing attention. Meanwhile, to promote the adaptability in multiple scenarios, other functions such as the thermal infrared stealth, hydrophobicity, heat insulation, thermal stability, and even wearability are gradually incorporated into the material development goals. Furthermore, combining with theoretical calculation to guide the design of EMW absorption materials is also well worth considered. In the overall view, it should be believed that the carbon-based MOFs derivatives can be regarded as one of the most promising materials for high-efficiency EMW absorption now and in the future.

## References

[1] Li X, Yin X, Song C, Han M, Xu H (2018). Self-assembly core–shell graphene-bridged hollow mxenes spheres 3D foam with ultrahigh specific em absorption performance. Adv. Funct. Mater..

[2] Chen W, Liu LX, Zhang HB, Yu ZZ (2020). Flexible, transparent, and conductive Ti_3_C_2_T_x_ MXene-silver nanowire films with smart acoustic sensitivity for high-performance electromagnetic interference shielding. ACS Nano.

[3] Namai A, Sakurai S, Nakajima M, Suemoto T, Matsumoto K (2009). Synthesis of an electromagnetic wave absorber for high-speed wireless communication. J. Am. Chem. Soc..

[4] Liu J, Zhang H-B, Sun R, Liu Y, Liu Z (2017). Hydrophobic, flexible, and lightweight MXene foams for high-performance electromagnetic-interference shielding. Adv. Mater..

[5] Zhao B, Shao G, Fan B, Zhao W, Xie Y (2015). Synthesis of flower-like cus hollow microspheres based on nanoflakes self-assembly and their microwave absorption properties. J. Mater. Chem. A.

[6] Zhang Y, Huang Y, Zhang T, Chang H, Xiao P (2015). Broadband and tunable high-performance microwave absorption of an ultralight and highly compressible graphene foam. Adv. Mater..

[7] Liu Q, Cao Q, Bi H, Liang C, Yuan K (2016). CoNi@SiO_2_@TiO_2_ and CoNi@air@TiO_2_ microspheres with strong wideband microwave absorption. Adv. Mater..

[8] Wang F, Wang N, Han X, Liu D, Wang Y (2019). Core–shell FeCo@carbon nanoparticles encapsulated in polydopamine-derived carbon nanocages for efficient microwave absorption. Carbon.

[9] Lv H, Yang Z, Wang PL, Ji G, Song J (2018). A voltage-boosting strategy enabling a low-frequency, flexible electromagnetic wave absorption device. Adv. Mater..

[10] Novoselov KS, Geim AK, Morozov SV, Jiang D, Katsnelson MI (2005). Two-dimensional gas of massless dirac fermions in graphene. Nature.

[11] De Volder MFL, Tawfick SH, Baughman RH, Hart AJ (2013). Carbon nanotubes: Present and future commercial applications. Science.

[12] Sun H, Che R, You X, Jiang Y, Yang Z (2014). Cross-stacking aligned carbon-nanotube films to tune microwave absorption frequencies and increase absorption intensities. Adv. Mater..

[13] Li Y, Liu X, Nie X, Yang W, Wang Y (2019). Multifunctional organic–inorganic hybrid aerogel for self-cleaning, heat-insulating, and highly efficient microwave absorbing material. Adv. Funct. Mater..

[14] Qiao J, Zhang X, Xu D, Kong L, Lv L (2020). Design and synthesis of TiO_2_/Co/carbon nanofibers with tunable and efficient electromagnetic absorption. Chem. Eng. J..

[15] Liu D, Du Y, Xu P, Wang F, Wang Y (2021). Rationally designed hierarchical N-doped carbon nanotubes wrapping waxberry-like Ni@C microspheres for efficient microwave absorption. J. Mater. Chem. A.

[16] Han B, Chu W, Han X, Xu P, Liu D (2020). Dual functions of glucose induced composition-controllable Co/C microspheres as high-performance microwave absorbing materials. Carbon.

[17] Wang L, Li X, Li Q, Yu X, Zhao Y (2019). Oriented polarization tuning broadband absorption from flexible hierarchical ZnO arrays vertically supported on carbon cloth. Small.

[18] Xia T, Zhang C, Oyler NA, Chen X (2013). Hydrogenated TiO_2_ nanocrystals: A novel microwave absorbing material. Adv. Mater..

[19] Ye F, Song Q, Zhang Z, Li W, Zhang S (2018). Direct growth of edge-rich graphene with tunable dielectric properties in porous Si_3_N_4_ ceramic for broadband high-performance microwave absorption. Adv. Funct. Mater..

[20] You W, Bi H, She W, Zhang Y, Che R (2017). Dipolar-distribution cavity gamma-Fe_2_O_3_@C@alpha-MnO_2_ nanospindle with broadened microwave absorption bandwidth by chemically etching. Small.

[21] Cui L, Wang Y, Han X, Xu P, Wang F (2021). Phenolic resin reinforcement: A new strategy for hollow NiCo@C microboxes against electromagnetic pollution. Carbon.

[22] Deng B, Liu Z, Pan F, Xiang Z, Zhang X (2021). Electrostatically self-assembled two-dimensional magnetized mxene/hollow Fe_3_O_4_ nanoparticle hybrids with high electromagnetic absorption performance and improved impendence matching. J. Mater. Chem. A.

[23] Eddaoudi M, Kim J, Rosi N, Vodak D, Wachter J (2002). Systematic design of pore size and functionality in isoreticular MOFs and their application in methane storage. Science.

[24] Kirchon A, Feng L, Drake HF, Joseph EA, Zhou H-C (2018). From fundamentals to applications: a toolbox for robust and multifunctional mof materials. Chem. Soc. Rev..

[25] Rowsell JLC, Yaghi OM (2004). Metal–organic frameworks: A new class of porous materials. Micropor. Mesopor. Mater..

[26] Mohamed E, Jaheon K, Nathaniel R, David V, Joseph W (2002). Systematic design of pore size and functionality in isoreticular MOFs and their application in methane storage. Science.

[27] Serre C, Millange F, Thouvenot C, Nogues M, Marsolier G (2002). Very large breathing effect in the first nanoporous chromium(III)-based solids: MIL-53 or Cr^III^(OH)·{O_2_C-C_6_H_4_-CO_2_}·{HO_2_C-C_6_H_4_-CO_2_H}_x_·H_2_O_y_. J. Am. Chem. Soc..

[28] Kondo M, Okubo T, Asami A, Noro S, Yoshitomi T (1999). Rational synthesis of stable channel-like cavities with methane gas adsorption properties: [{Cu_2_(pzdc)_2_(L)}_n_] (pzdc=pyrazine-2,3-dicarboxylate; L=a Pillar Ligand). Angew. Chem. Int. Ed..

[29] Wang B, Côté AP, Furukawa H, O’Keeffe M, Yaghi OM (2008). Colossal cages in zeolitic imidazolate frameworks as selective carbon dioxide reservoirs. Nature.

[30] Ma S, Zhou H-C (2006). A metal-organic framework with entatic metal centers exhibiting high gas adsorption affinity. J. Am. Chem. Soc..

[31] Cavka JH, Jakobsen S, Olsbye U, Guillou N, Lamberti C (2008). A new zirconium inorganic building brick forming metal organic frameworks with exceptional stability. J. Am. Chem. Soc..

[32] Thompson JA, Blad CR, Brunelli NA, Lydon ME, Lively RP (2012). Hybrid zeolitic imidazolate frameworks: controlling framework porosity and functionality by mixed-linker synthesis. Chem. Mater..

[33] Su Z, Fan J, Okamura T-A, Sun W-Y, Ueyama N (2010). Ligand-directed and ph-controlled assembly of chiral 3d–3d heterometallic metal−organic frameworks. Cryst. Growth Des..

[34] Yang H, Wang X (2018). Secondary-component incorporated hollow MOFs and derivatives for catalytic and energy-related applications. Adv. Mater..

[35] Hou C-C, Xu Q (2019). Metal–organic frameworks for energy. Adv. Energy Mater..

[36] Rocca DJ, Liu D, Lin W (2011). Nanoscale metal–organic frameworks for biomedical imaging and drug delivery. Accounts Chem. Res..

[37] Gu Z-Y, Yang C-X, Chang N, Yan X-P (2012). Metal–organic frameworks for analytical chemistry: from sample collection to chromatographic separation. Accounts Chem. Res..

[38] Li J-R, Sculley J, Zhou H-C (2012). Metal-organic frameworks for separations. Chem. Rev..

[39] Dang S, Zhu Q-L, Xu Q (2018). Nanomaterials derived from metal–organic frameworks. Nat. Rev. Mater..

[40] Qiang R, Du Y, Zhao H, Wang Y, Tian C (2015). Metal organic framework-derived Fe/C nanocubes toward efficient microwave absorption. J. Mater. Chem. A.

[41] Cao X, Tan C, Sindoro M, Zhang H (2017). Hybrid micro-/nano-structures derived from metal–organic frameworks: Preparation and applications in energy storage and conversion. Chem. Soc. Rev..

[42] Yang Z, Lv H, Wu R (2016). Rational construction of graphene oxide with MOF-derived porous NiFe@C nanocubes for high-performance microwave attenuation. Nano Res..

[43] Zhang X, Qiao J, Liu C, Wang F, Jiang Y (2020). A MOF-derived ZrO_2_/C nanocomposite for efficient electromagnetic wave absorption. Inorg. Chem. Front..

[44] Ma J, Zhang X, Liu W, Ji G (2016). Direct synthesis of MOF-derived nanoporous CuO/carbon composites for high impedance matching and advanced microwave absorption. J. Mater. Chem. C.

[45] Lu Y, Wang Y, Li H, Lin Y, Jiang Z (2015). MOF-derived porous Co/C nanocomposites with excellent electromagnetic wave absorption properties. ACS Appl. Mater. Interfaces.

[46] Yin Y, Liu X, Wei X, Li Y, Nie X (2017). Magnetically aligned Co-C/MWCNTs composite derived from mwcnt-interconnected zeolitic imidazolate frameworks for a lightweight and highly efficient electromagnetic wave absorber. ACS Appl. Mater. Interfaces.

[47] Qiu Y, Lin Y, Yang H, Wang L, Wang M (2020). Hollow Ni/C microspheres derived from Ni-metal organic framework for electromagnetic wave absorption. Chem. Eng. J..

[48] Zhang X, Ji G, Liu W, Zhang X, Gao Q (2016). A novel Co/TiO_2_ nanocomposite derived from a metal–organic framework: Synthesis and efficient microwave absorption. J. Mater. Chem. C.

[49] Wang L, Yu X, Li X, Zhang J, Wang M (2020). MOF-derived yolk–shell Ni@C@ZnO schottky contact structure for enhanced microwave absorption. Chem. Eng. J..

[50] Liao Q, He M, Zhou Y, Nie S, Wang Y (2018). Highly cuboid-shaped heterobimetallic metal–organic frameworks derived from porous Co/ZnO/C microrods with improved electromagnetic wave absorption capabilities. ACS Appl. Mater. Interfaces.

[51] Liao Q, He M, Zhou Y, Nie S, Wang Y (2018). Rational construction of Ti_3_C_2_T_x_/Co-MOF-derived laminated Co/TiO_2_-C hybrids for enhanced electromagnetic wave absorption. Langmuir.

[52] Liu W, Tan S, Yang Z, Ji G (2018). Hollow graphite spheres embedded in porous amorphous carbon matrices as lightweight and low-frequency microwave absorbing material through modulating dielectric loss. Carbon.

[53] Xu H, Yin X, Zhu M, Li M, Zhang H (2019). Constructing hollow graphene nano-spheres confined in porous amorphous carbon particles for achieving full x band microwave absorption. Carbon.

[54] C.M. Watts, X. Liu, W.J. Padilla, Metamaterial electromagnetic wave absorbers. Adv. Mater. **24**, OP98–OP120 (2012). 10.1002/adma.201200674 10.1002/adma.201200674 22627995

[55] Thomassin J-M, Lou X, Pagnoulle C, Saib A, Bednarz L (2007). Multiwalled carbon nanotube/poly(epsilon-caprolactone) nanocomposites with exceptional electromagnetic interference shielding properties. J. Phys. Chem. C.

[56] Chen H, Ma W, Huang Z, Zhang Y, Huang Y (2019). Graphene-based materials toward microwave and terahertz absorbing stealth technologies. Adv. Opt. Mater..

[57] Li Q, Zhang Z, Qi L, Liao Q, Kang Z (2019). Toward the application of high frequency electromagnetic wave absorption by carbon nanostructures. Adv. Sci..

[58] Pan F, Liu Z, Deng B, Dong Y, Zhu X (2021). Lotus leaf-derived gradient hierarchical porous C/MoS_2_ morphology genetic composites with wideband and tunable electromagnetic absorption performance. Nano-Micro Lett..

[59] Meshram MR, Agrawal NK, Sinha B, Misra PS (2004). Characterization of m-type barium hexagonal ferrite-based wide band microwave absorber. J. Magn. Magn. Mater..

[60] Wang Y, Li X, Han X, Xu P, Cui L (2020). Ternary Mo_2_C/Co/C composites with enhanced electromagnetic waves absorption. Chem. Eng. J..

[61] Naito Y, Suetake K (1971). Application of ferrite to electromagnetic wave absorber and its characteristic. IEEE T. Microw. Theory Techn..

[62] Kim SS, Jo SB, Gueon KI, Choi KK, Kim JM (1991). Complex permeability and permittivity and microwave absorption of ferrite-rubber composite in X-band frequencies. IEEE T. Magn..

[63] Blokhintzev D (1946). The propagation of sound in an inhomogeneous and moving medium I. J. Acoust. Soc. Am..

[64] Liang C, Liu C, Wang H, Wu L, Jiang Z (2014). SiC–Fe_3_O_4_ dielectric–magnetic hybrid nanowires: controllable fabrication, characterization and electromagnetic wave absorption. J. Mater. Chem. A.

[65] Ma Z, Cao C-T, Liu Q-F, Wang J-B (2012). A new method to calculate the degree of electromagnetic impedance matching in one-layer microwave absorbers. Chin. Phys. Lett..

[66] Huang Y-Q, Yuan J, Song W-L, Wen B, Fang X-Y (2010). Microwave absorbing materials: solutions for real functions under ideal conditions of microwave absorption. Chin. Phys. Lett..

[67] Kong L, Yin X, Zhang Y, Yuan X, Li Q (2013). Electromagnetic wave absorption properties of reduced graphene oxide modified by maghemite colloidal nanoparticle clusters. J. Phys. Chem. C.

[68] Kong L, Yin X, Ye F, Li Q, Zhang L (2013). Electromagnetic wave absorption properties of ZnO-based materials modified with ZnAl_2_O_4_ nanograins. J. Phys. Chem. C.

[69] Shirakata Y, Hidaka N, Ishitsuka M, Teramoto A, Ohmi T (2008). High permeability and low loss Ni–Fe composite material for high-frequency applications. IEEE Trans. Magn..

[70] Inui T, Konishi K (1999). Fabrications of broad-band RF-absorber composed of planar hexagonal ferrites. IEEE Trans. Magn..

[71] Yu Z, Zhang N, Yao Z, Han X, Jiang Z (2013). Synthesis of hierarchical dendritic micro–nano structure Co_x_Fe_1−x_ alloy with tunable electromagnetic absorption performance. J. Mater. Chem. A.

[72] Qi X, Yang Y, Zhong W, Qin C, Deng Y (2010). Simultaneous synthesis of carbon nanobelts and carbon/Fe–Cu hybrids for microwave absorption. Carbon.

[73] Olmedo L, Hourquebie P, Jousse F (1993). Microwave absorbing materials based on conducting polymers. Adv. Mater..

[74] Liu P, Gao S, Wang Y, Huang Y, Wang Y (2019). Core–shell CoNi@graphitic carbon decorated on B, N-codoped hollow carbon polyhedrons toward lightweight and high-efficiency microwave attenuation. ACS Appl. Mater. Interfaces.

[75] Deng LJ, Han MG (2007). Microwave absorbing performances of multiwalled carbon nanotube composites with negative permeability. Appl. Phys. Lett..

[76] Liang L, Li Q, Yan X, Feng Y, Wang Y (2021). Multifunctional magnetic Ti_3_C_2_T_x_ mxene/graphene aerogel with superior electromagnetic wave absorption performance. ACS Nano.

[77] Xu H, Yin X, Zhu M, Han M, Hou Z (2017). Carbon hollow microspheres with a designable mesoporous shell for high-performance electromagnetic wave absorption. ACS Appl. Mater. Interfaces.

[78] Yan F, Zhang S, Zhang X, Li C, Zhu C (2018). Growth of CoFe_2_O_4_ hollow nanoparticles on graphene sheets for high-performance electromagnetic wave absorbers. J. Mater. Chem. C.

[79] Wang L, Xing H, Gao S, Ji X, Shen Z (2017). Porous flower-like NiO@graphene composites with superior microwave absorption properties. J. Mater. Chem. C.

[80] Xu Z, Du Y, Liu D, Wang Y, Ma W (2019). Pea-like Fe/Fe_3_C nanoparticles embedded in nitrogen-doped carbon nanotubes with tunable dielectric/magnetic loss and efficient electromagnetic absorption. ACS Appl. Mater. Interfaces.

[81] Dong S, Zhang W, Zhang X, Hu P, Han J (2018). Designable synthesis of core–shell SiCw@C heterostructures with thickness-dependent electromagnetic wave absorption between the whole X-band and Ku-band. Chem. Eng. J..

[82] Cole KS, Cole HR (1941). Dispersion and absorption in dielectrics I. Alternating current characteristics. J. Chem. Phys..

[83] Zhdanov M (2008). Generalized effective-medium theory of induced polarization. Geophysics.

[84] Wang C, Han XJ, Xu P, Zhang XL, Du YC (2011). The electromagnetic property of chemically reduced graphene oxide and its application as microwave absorbing material. Appl. Phys. Lett..

[85] Dong S, Song J, Zhang X, Hu P, Sun B (2017). Strong contribution of in situ grown nanowires to enhance the thermostabilities and microwave absorption properties of porous graphene foams under different atmospheres. J. Mater. Chem. C..

[86] Qiao J, Zhang X, Liu C, Lyu L, Yang Y (2021). Non-magnetic bimetallic MOF-derived porous carbon-wrapped TiO_2_/ZrTiO_4_ composites for efficient electromagnetic wave absorption. Nano-Micro Lett..

[87] Lu B, Dong XL, Huang H, Zhang XF, Zhu XG (2008). Microwave absorption properties of the core/shell-type iron and nickel nanoparticles. J. Magn. Magn. Mater..

[88] Li D, Liao H, Kikuchi H, Liu T (2017). Microporous Co@C nanoparticles prepared by dealloying CoAl@C precursors: Achieving strong wideband microwave absorption via controlling carbon shell thickness. ACS Appl. Mater. Interfaces.

[89] Wu MZ, Zhang YD, Hui S, Xiao TD, Ge S (2002). Microwave magnetic properties of Co_50_/(SiO_2_)_50_ nanoparticles. Appl. Phys. Lett..

[90] Qiao J, Zhang X, Liu C, Lyu L, Wang Z (2020). Facile fabrication of Ni embedded TiO_2_/C core–shell ternary nanofibers with multicomponent functional synergy for efficient electromagnetic wave absorption. Compos. B Eng..

[91] Zhou N, An Q, Xiao Z, Zhai S, Shi Z (2017). Rational design of superior microwave shielding composites employing synergy of encapsulating character of alginate hydrogels and task-specific components (Ni NPs, Fe_3_O_4_/CNTs). ACS Sustain. Chem. Eng..

[92] Kittel C (1948). On the theory of ferromagnetic resonance absorption. Phys. Rev..

[93] Jian X, Wu B, Wei Y, Dou SX, Wang X (2016). Facile synthesis of Fe_3_O_4_/GCs composites and their enhanced microwave absorption properties. ACS Appl. Mater. Interfaces.

[94] Zhang XF, Dong XL, Huang H, Liu YY, Wang WN (2006). Microwave absorption properties of the carbon-coated nickel nanocapsules. Appl. Phys. Lett..

[95] Zhao H, Cheng Y, Liu W, Yang Z, Zhang B (2018). The flaky porous Fe_3_O_4_ with tunable dimensions for enhanced microwave absorption performance in X and C bands. Nanotechnology.

[96] Zhao Y, Liu L, Han J, Wu W, Tong G (2017). Effective modulation of electromagnetic characteristics by composition and size in expanded graphite/Fe_3_O_4_ nanoring composites with high snoek's limit. J. Alloys Compd..

[97] Bush GG (1988). Generalization of Snoek’s limit for modeling initial permeability of magnetic materials. J. Appl. Phys..

[98] Huang L, Li J, Wang Z, Li Y, He X (2019). Microwave absorption enhancement of porous C@CoFe_2_O_4_ nanocomposites derived from eggshell membrane. Carbon.

[99] Xu W, Wang G-S, Yin P-G (2018). Designed fabrication of reduced graphene oxides/Ni hybrids for effective electromagnetic absorption and shielding. Carbon.

[100] Feng J, Hou Y, Wang Y, Li L (2017). Synthesis of hierarchical ZnFe_2_O_4_@SiO_2_@rGO core–shell microspheres for enhanced electromagnetic wave absorption. ACS Appl. Mater. Interfaces.

[101] Wu N, Liu C, Xu D, Liu J, Liu W (2018). Enhanced electromagnetic wave absorption of three-dimensional porous Fe_3_O_4_/C composite flowers. ACS Sustain. Chem. Eng..

[102] Zhao H, Xu X, Wang Y, Fan D, Liu D (2020). Heterogeneous interface induced the formation of hierarchically hollow carbon microcubes against electromagnetic pollution. Small.

[103] Ma J, Liu W, Liang X, Quan B, Cheng Y (2017). Nanoporous TiO2/C composites synthesized from directly pyrolysis of a Ti-based MOFs MIL-125(Ti) for efficient microwave absorption. J. Alloys Compd..

[104] Wu Q, Jin H, Chen W, Huo S, Chen X (2018). Graphitized nitrogen-doped porous carbon composites derived from ZIF-8 as efficient microwave absorption materials. Mater. Res. Express.

[105] Huang L, Huang S, Yang Z, Zhao A, Liu C (2018). In-situ conversion of ZnO/Ni3ZnC0.7/CNT composite from NiZn bimetallic MOF precursor with enhanced electromagnetic property. Nanomaterials.

[106] Liang X, Quan B, Ji G, Liu W, Zhao H (2017). Tunable dielectric performance derived from the metal–organic framework/reduced graphene oxide hybrid with broadband absorption. ACS Sustain. Chem. Eng..

[107] Dai S, Cheng Y, Quan B, Liang X, Liu W (2018). Porous-carbon-based Mo_2_C nanocomposites as excellent microwave absorber: A new exploration. Nanoscale.

[108] Quan B, Liang X, Yi H, Chen Y, Xiang J (2018). Thermal conversion of wheat-like metal organic frameworks to achieve MgO/carbon composites with tunable morphology and microwave response. J. Mater. Chem. C.

[109] Yan J, Huang Y, Han X, Gao X, Liu P (2019). Metal organic framework (ZIF-67)-derived hollow CoS_2_/N-doped carbon nanotube composites for extraordinary electromagnetic wave absorption. Compos. B Eng..

[110] Liu P, Zhu C, Gao S, Guan C, Huang Y (2020). N-doped porous carbon nanoplates embedded with CoS_2_ vertically anchored on carbon cloths for flexible and ultrahigh microwave absorption. Carbon.

[111] Wang K, Chen Y, Tian R, Li H, Zhou Y (2018). Porous Co-C core–shell nanocomposites derived from Co-MOF-74 with enhanced electromagnetic wave absorption performance. ACS Appl. Mater. Interfaces.

[112] Huang L, Liu X, Yu R (2018). An efficient Co/C microwave absorber with tunable Co nanoparticles derived from a ZnCo bimetallic zeolitic imidazolate framework. Part. Part. Syst. Char..

[113] Zhu B-Y, Miao P, Kong J, Zhang X-L, Wang G-Y (2019). Co/C composite derived from a newly constructed metal–organic framework for effective microwave absorption. Cryst. Growth Des..

[114] Li J, Miao P, Chen K-J, Cao J-W, Liang J (2020). Highly effective electromagnetic wave absorbing prismatic Co/C nanocomposites derived from cubic metal–organic framework. Compos. B Eng..

[115] Xiao X, Zhu W, Tan Z, Tian W, Guo Y (2018). Ultra-small Co/CNTs nanohybrid from metal organic framework with highly efficient microwave absorption. Compos. B Eng..

[116] Li Z, Han X, Ma Y, Liu D, Wang Y (2018). MOFs-derived hollow Co/C microspheres with enhanced microwave absorption performance. ACS Sustain. Chem. Eng..

[117] Wang H, Xiang L, Wei W, An J, He J (2017). Efficient and lightweight electromagnetic wave absorber derived from metal organic framework-encapsulated cobalt nanoparticles. ACS Appl. Mater. Interfaces.

[118] Liu W, Tan S, Yang Z, Ji G (2018). Enhanced low-frequency electromagnetic properties of MOF-derived cobalt through interface design. ACS Appl. Mater. Interfaces.

[119] Gu W, Tan J, Chen J, Zhang Z, Zhao Y (2020). Multifunctional bulk hybrid foam for infrared stealth, thermal insulation, and microwave absorption. ACS Appl. Mater. Interfaces.

[120] Yang N, Luo Z-X, Zhu G-R, Chen S-C, Wang X-L (2019). Ultralight three-dimensional hierarchical cobalt nanocrystals/N-doped CNTs/carbon sponge composites with a hollow skeleton toward superior microwave absorption. ACS Appl. Mater. Interfaces.

[121] Yang ML, Yuan Y, Li Y, Sun XX, Wang SS (2020). Dramatically enhanced electromagnetic wave absorption of hierarchical CNT/Co/C fiber derived from cotton and metal–organic-framework. Carbon.

[122] Wang L, Bai X, Wen B, Du Z, Lin Y (2019). Honeycomb-like Co/C composites derived from hierarchically nanoporous ZIF-67 as a lightweight and highly efficient microwave absorber. Compos. B. Eng..

[123] Xu X, Ran F, Fan Z, Lai H, Cheng Z (2019). Cactus-inspired bimetallic metal–organic framework-derived 1D–2D hierarchical Co/N-decorated carbon architecture toward enhanced electromagnetic wave absorbing performance. ACS Appl. Mater. Interfaces.

[124] Shu R, Li W, Wu Y, Zhang J, Zhang G (2019). Nitrogen-doped Co-C/MWCNTs nanocomposites derived from bimetallic metal–organic frameworks for electromagnetic wave absorption in the X-band. Chem. Eng. J..

[125] Feng W, Wang Y, Chen J, Li B, Guo L (2018). Metal organic framework-derived CoZn alloy/N-doped porous carbon nanocomposites: Tunable surface area and electromagnetic wave absorption properties. J. Mater. Chem. C.

[126] Wu N, Xu D, Wang Z, Wang F, Liu J (2019). Achieving superior electromagnetic wave absorbers through the novel metal–organic frameworks derived magnetic porous carbon nanorods. Carbon.

[127] Miao P, Zhou R, Chen K, Liang J, Ban Q (2020). Tunable electromagnetic wave absorption of supramolecular isomer-derived nanocomposites with different morphology. Adv. Mater. Interfaces.

[128] Liu Q, Liu X, Feng H, Shui H, Yu R (2017). Metal organic framework-derived Fe/carbon porous composite with low fe content for lightweight and highly efficient electromagnetic wave absorber. Chem. Eng. J..

[129] Liu W, Shao Q, Ji G, Liang X, Cheng Y (2017). Metal–organic-frameworks derived porous carbon-wrapped Ni composites with optimized impedance matching as excellent lightweight electromagnetic wave absorber. Chem. Eng. J..

[130] Yan J, Huang Y, Yan Y, Ding L, Liu P (2019). High-performance electromagnetic wave absorbers based on two kinds of nickel-based MOF-derived Ni@C microspheres. ACS Appl. Mater. Interfaces.

[131] Liu D, Du Y, Xu P, Liu N, Wang Y (2019). Waxberry-like hierarchical Ni@C microspheres with high-performance microwave absorption. J. Mater. Chem. C.

[132] Wang L, Wen B, Yang H, Qiu Y, He N (2020). Hierarchical nest-like structure of Co/Fe MOF derived CoFe@C composite as wide-bandwidth microwave absorber. Compos. Part A Appl. Sci. Manuf..

[133] Zhang X, Ji G, Liu W, Quan B, Liang X (2015). Thermal conversion of an Fe_3_O_4_@metal–organic framework: A new method for an efficient Fe–Co/nanoporous carbon microwave absorbing material. Nanoscale.

[134] Wang S, Xu Y, Fu R, Zhu H, Jiao Q (2019). Rational construction of hierarchically porous Fe–Co/N-doped carbon/rGO composites for broadband microwave absorption. Nano-Micro Lett..

[135] Xiong Y, Xu L, Yang C, Sun Q, Xu X (2020). Implanting FeCo/C nanocages with tunable electromagnetic parameters in anisotropic wood carbon aerogels for efficient microwave absorption. J. Mater. Chem. A.

[136] Wang Y, Wang H, Ye J, Shi L, Feng X (2020). Magnetic CoFe alloy@C nanocomposites derived from ZnCo-MOF for electromagnetic wave absorption. Chem. Eng. J..

[137] Wang Y-L, Yang S-H, Wang H-Y, Wang G-S, Sun X-B (2020). Hollow porous CoNi/C composite nanomaterials derived from MOFs for efficient and lightweight electromagnetic wave absorber. Carbon.

[138] Xu X, Ran F, Fan Z, Cheng Z, Lv T (2020). Bimetallic metal–organic framework-derived pomegranate-like nanoclusters coupled with CoNi-doped graphene for strong wideband microwave absorption. ACS Appl. Mater. Interfaces.

[139] Wang L, Huang M, Yu X, You W, Zhang J (2020). MOF-derived Ni_1−x_Co_x_@carbon with tunable nano–microstructure as lightweight and highly efficient electromagnetic wave absorber. Nano-Micro Lett..

[140] Xiong J, Xiang Z, Zhao J, Yu L, Cui E (2019). Layered nico alloy nanoparticles/nanoporous carbon composites derived from bimetallic MOFs with enhanced electromagnetic wave absorption performance. Carbon.

[141] Xu X, Ran F, Lai H, Cheng Z, Lv T (2019). In situ confined bimetallic metal–organic framework derived nanostructure within 3D interconnected bamboo-like carbon nanotube networks for boosting electromagnetic wave absorbing performances. ACS Appl. Mater. Interfaces.

[142] Ouyang J, He Z, Zhang Y, Yang H, Zhao Q (2019). Trimetallic FeCoNi@C nanocomposite hollow spheres derived from metal–organic frameworks with superior electromagnetic wave absorption ability. ACS Appl. Mater. Interfaces.

[143] Xiang Z, Song Y, Xiong J, Pan Z, Wang X (2019). Enhanced electromagnetic wave absorption of nanoporous Fe_3_O_4_@carbon composites derived from metal–organic frameworks. Carbon.

[144] Liu W, Liu L, Ji G, Li D, Zhang Y (2017). Composition design and structural characterization of MOF-derived composites with controllable electromagnetic properties. ACS Sustain. Chem. Eng..

[145] Deng B, Xiang Z, Xiong J, Liu Z, Yu L (2020). Sandwich-like Fe&TiO_2_@C nanocomposites derived from MXene/Fe-MOFs hybrids for electromagnetic absorption. Nano-Micro Lett..

[146] Zhou C, Wu C, Liu D, Yan M (2018). Metal–organic framework derived hierarchical Co/C@V_2_O_3_ hollow spheres as a thin, lightweight and high-efficiency electromagnetic wave absorber. Chem. Eur. J..

[147] Liang X, Quan B, Ji G, Liu W, Cheng Y (2016). Novel nanoporous carbon derived from metal–organic frameworks with tunable electromagnetic wave absorption capabilities. Inorg. Chem. Front..

[148] Feng W, Wang Y, Zou Y, Chen J, Jia D (2018). ZnO@N-doped porous carbon/Co_3_ZnC core–shell heterostructures with enhanced electromagnetic wave attenuation ability. Chem. Eng. J..

[149] Liu W, Liu L, Yang Z, Xu J, Hou Y (2018). A versatile route toward the electromagnetic functionalization of metal–organic framework-derived three-dimensional nanoporous carbon composites. ACS Appl. Mater. Interfaces.

[150] Huang L, Chen C, Huang X, Ruan S, Zeng Y-J (2019). Enhanced electromagnetic absorbing performance of MOF-derived Ni/NiO/Cu@C composites. Compos. B Eng..

[151] Zhang X, Qiao J, Zhao J, Xu D, Wang F (2019). High-efficiency electromagnetic wave absorption of cobalt-decorated NH_2_-UiO-66-derived porous ZrO_2_/C. ACS Appl. Mater. Interfaces.

[152] Wang R, He M, Zhou Y, Nie S, Wang Y (2020). Metal−organic frameworks self-templated cubic hollow Co/N/C@MnO_2_ composites for electromagnetic wave absorption. Carbon.

[153] Zhang Y, Yang Z, Li M, Yang L, Liu J (2020). Heterostructured CoFe@C@MnO_2_ nanocubes for efficient microwave absorption. Chem. Eng. J..

[154] Zhao Z, Xu S, Du Z, Jiang C, Huang X (2019). Metal–organic framework-based Pb@MoS_2_ core–shell microcubes with high efficiency and broad bandwidth for microwave absorption performance. ACS Sustain. Chem. Eng..

[155] Liu X, Hao C, He L, Yang C, Chen Y (2018). Yolk–shell structured Co-C/void/Co_9_S_8_ composites with a tunable cavity for ultrabroadband and efficient low-frequency microwave absorption. Nano Res..

[156] Zhang K, Wu F, Xie A, Sun M, Dong W (2017). In situ stringing of metal organic frameworks by SiC nanowires for high-performance electromagnetic radiation elimination. ACS Appl. Mater. Interfaces.

[157] Xu H, Yin X, Li M, Ye F, Han M (2018). Mesoporous carbon hollow microspheres with red blood cell like morphology for efficient microwave absorption at elevated temperature. Carbon.

[158] Feng W, Wang Y, Chen J, Wang L, Guo L (2016). Reduced graphene oxide decorated with in-situ growing zno nanocrystals: Facile synthesis and enhanced microwave absorption properties. Carbon.

[159] Lyulyukin MN, Kolinko PA, Selishchev DS, Kozlov DV (2018). Hygienic aspects of TiO_2_-mediated photocatalytic oxidation of volatile organic compounds: Air purification analysis using a total hazard index. Appl. Catal. B Environ..

[160] Wu N, Lv H, Liu J, Liu Y, Wang S (2016). Improved electromagnetic wave absorption of Co nanoparticles decorated carbon nanotubes derived from synergistic magnetic and dielectric losses. Phys. Chem. Chem. Phys..

[161] Man Z, Li P, Zhou D, Wang Y, Liang X (2020). Two birds with one stone: FeS_2_@C yolk–shell composite for high-performance sodium-ion energy storage and electromagnetic wave absorption. Nano Lett..

[162] Mebarki M, Layadi A, Guittoum A, Benabbas A, Ghebouli B (2011). Structural and electrical properties of evaporated fe thin films. Appl. Surf. Sci..

[163] Zhao B, Guo X, Zhao W, Deng J, Shao G (2016). Yolk–shell Ni@SnO_2_ composites with a designable interspace to improve the electromagnetic wave absorption properties. ACS Appl. Mater. Interfaces.

[164] Wang L, Li X, Li Q, Zhao Y, Che R (2018). Enhanced polarization from hollow cube-like ZnSnO_3_ wrapped by multiwalled carbon nanotubes: As a lightweight and high-performance microwave absorber. ACS Appl. Mater. Interfaces.

[165] Zhao B, Guo X, Zhao W, Deng J, Fan B (2017). Facile synthesis of yolk–shell Ni@void@SnO_2_(Ni_3_Sn_2_) ternary composites via galvanic replacement/kirkendall effect and their enhanced microwave absorption properties. Nano Res..

[166] Lv W, Mei Q, Xiao J, Du M, Zheng Q (2017). 3D multiscale superhydrophilic sponges with delicately designed pore size for ultrafast oil/water separation. Adv. Funct. Mater..

[167] Wang L, Guan Y, Qiu X, Zhu H, Pan S (2017). Efficient ferrite/Co/porous carbon microwave absorbing material based on ferrite@metal–organic framework. Chem. Eng. J..

